# A luminescent view of the clickable assembly of LnF_3_ nanoclusters

**DOI:** 10.1038/s41467-021-23176-y

**Published:** 2021-05-19

**Authors:** Jie Zhou, Yang Wei, Yue Pan, Yue Wang, Ze Yuan, Fan Zhang, Hao Song, Jingyi Yue, Haiquan Su, Xiaoji Xie, Ling Huang

**Affiliations:** 1 Institute of Advanced Materials (IAM), Nanjing Tech University, 30 South Puzhu Road, Nanjing, China; 2 School of Chemistry and Chemical Engineering, Inner Mongolia University, 235 West Daxue Road, Hohhot, China

**Keywords:** Inorganic chemistry, Materials for optics, Molecular self-assembly

## Abstract

Nanoclusters (NCs) bridge the gap between atoms and nanomaterials in not only dimension but also physicochemical properties. Precise chemical and structural control, as well as clear understanding of formation mechanisms, have been important to fabricate NCs with high performance in optoelectronics, catalysis, nanoalloys, and energy conversion and harvesting. Herein, taking advantage of the close chemical properties of Ln^3+^ (Ln = Eu, Nd, Sm, Gd, etc.) and Gd^3+^–Eu^3+^ energy transfer ion-pair, we report a clickable LnF_3_ nanoparticle assembly strategy allowing reliable fabrication of diversely structured NCs, including single-component, dimeric, core-shelled/core-shell-shelled, and reversely core-shelled/core-shell-shelled, particularly with synergized optical functionalities. Moreover, the purposely-embedded dual luminescent probes offer great superiority for in situ and precise tracking of tiny structural variations and energy transfer pathways within complex nanoarchitectures.

## Abstract (web summary)

Precisely controlling the chemical composition and structure of nanoclusters is an ongoing challenge. Here, the authors report a clickable assembly strategy to construct widely varied lanthanide nanoclusters with synergized optical functionalities.

## Introduction

Organic ligands have played an irreplaceable role in myriads of nanomaterials synthesis towards control of size^1,2^, morphology^3,4^, crystal structure^5^, and functionality^6,7^. Meanwhile, rational selection of the chemical groups on ligand molecules is also critical for post-modulations such as assembly, hybridization, and fabrication of micro or macroscaled architectures from individual nanoparticles (NPs) aiming at unprecedented chemical and physical properties^8,9^, particularly when other technologies such as Langmuir-Blodgett^10,11^, layer-by-layer^12^, and optical or electron-beam lithography are combined^13,14^.

Moreover, development of a library of ligand exchange methods through nanoscale chemical reactions has facilitated easy conversion of nanomaterials from hydrophobic to hydrophilic or vice versa^15–17^, which generates additional functionalities to realize various purposes such as QLED display^18^, trace analysis^19^, disease diagnosis^20^, bioimaging^21^, as well as photodynamic therapy^22^, and so on. As a typical example, replacement of citrate ligand on gold NPs with thiol-ended single chain DNA has enabled successful assembly of gold NPs possessing complementary DNA sequences, which leads to precise fabrication of supercrystals with varied lattice structures^23^ and remarkable features that neither individual component possesses^24,25^.

However, ligand molecules may also affect or even prevent functionality expression. For example, insulating molecules are unwanted for semiconducting quantum dots when used for nanodevice fabrication^26^, chemical molecules surrounding perovskite nanocrystals usually exacerbate the optoelectronic performance in therein fabricated solar cells^27^, and catalytic noble metal NPs get poisoned by organic molecules used as protecting ligands and so do for the photo- and/or electrochemical catalysis^28^. On the other side, aggregation of nanomaterials occurs once ligands are removed, accompanied with irreversible functionality loss. Thus, it remains an urgent challenge to find an optimal balance between this dilemma, that is, how to fully exploit the functionalities of nanomaterials that are prone to aggregation once without ligand and meanwhile avoid the influence of ligand molecules, particularly for tiny NPs (<5 nm) that are very active due to the high surface energy^29^.

Herein, we have developed a clickable assembly strategy that facilitates reliable fabrication of variously structured lanthanide nanoclusters (NCs) with synergized multi-functionalities. The formation of LnF_3_ NCs starts with conversion of LnOF NPs to LnF_3_ NPs after organic ligand removal, followed by the assembly process. This HCl-triggered click-reaction allows arbitrary fabrication of the compositions and structures of target NCs such as single-component, dimeric, core-shelled/core-shell-shelled, and reversely core-shelled/core-shell-shelled. The embedded dual luminescent probes via either dynamic energy transfer (Gd^3+^–Eu^3+^) or direct excitation (of Eu^3+^) enables sensitive track of tiny variations in structure and energy transfer pathways.

## Results

### Assembly of EuOF NPs into EuF_3_ NCs

**Fig. 1:**
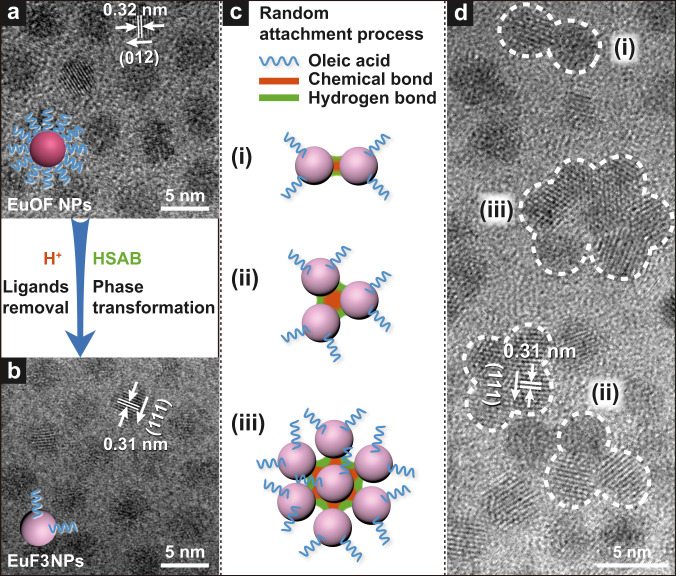
Self-assembly mechanism of NCs. High-resolution transmission electron microscope (HRTEM) images of **a** EuOF NPs and **b** EuF_3_ NPs. **c** Schematic diagram illustrating the structural details, random attachment process, and interaction forces responsible for NCs formation. **d** HRTEM image taken at assembly time of 15 min. The insets in **a** and **b** enlighten the difference of the number of ligand molecules after H^+^ introduction. TEM structures of (i), (ii), and (iii) in **d** correspond exactly to those in **c**.

Specifically, addition of certain amount of diluted HCl into the stock solution of EuOF NPs (Fig. 1a and Supplementary Fig. 1) strips off majority of the oleic acid anion (OA^−^) ligands and almost simultaneously triggers the conversion to small EuF_3_ NPs (Fig. 1b). The coordination between surface Eu^3+^ on EuF_3_ NPs and solvent molecules (H_2_O or C_2_H_5_OH)^30^ improves the solubility. Although these consequent nanoscaled-rare earth chemical reactions complete within 5 mins, they describe exactly why the solution changes from opaque to transparent after HCl addition (Supplementary Fig. 2a, b). However, EuF_3_ NPs are metastable due to high surface energy and finally assemble into stable NCs through a random attachment mechanism (Fig. 1c, d, Supplementary Fig. 3 and Supplementary Fig. 4). The reason we speculate that the final solution turned back from transparent to opaque (Supplementary Fig. 2c) is that, the trace amount of OA^−^ left on each EuF_3_ NP gradually accumulated on NCs surface when assembly progresses, which leads to increased hydrophobicity and thus decreased solubility in ethanol (Fig. 1c).

**Fig. 2:**
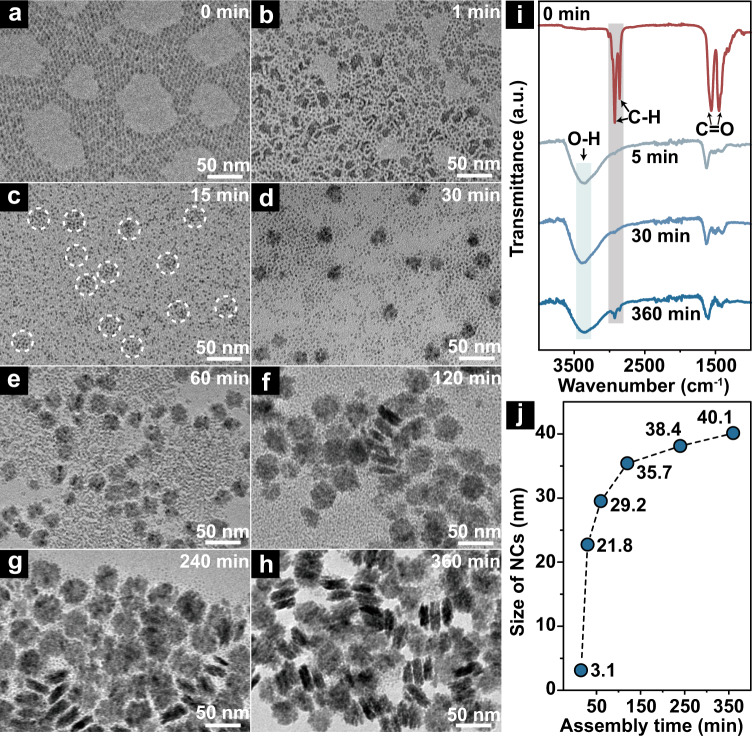
Different assembly stages of NCs. TEM images of the products obtained at assembly time of **a** 0, **b** 1, **c** 15, **d** 30, **e** 60, **f** 120, **g** 240, and **h** 360 min, respectively. **i** FT-IR spectra of the products obtained at the assembly time of 0, 5, 30, and 360 min, respectively. **j** Time-dependent size evolution of NCs. White dotted-circles in **c** show the NCs formed at very early stage.

Based on systematic analysis of TEM (Fig. 2a–h) and FT-IR (Fig. 2i) results of the samples collected at different assembly times, the growth process of EuF_3_ NCs can be divided into the following three stages:

Stage 1: Conversion of EuOF NPs to EuF_3_ NPs. This is the most critical step, which forms the foundation for subsequent assembly and growth of EuF_3_ NCs. TEM images (Fig. 2a–c and Supplementary Fig. 5) suggest that EuOF NPs at diameter of 3.2 nm turned into semi-dissociated morphologies after 1 min of H^+^ addition, which is caused by the ligands (OA^−^) removal induced by the post-added H^+^^30,31^. Then, chemical conversion of EuOF NPs to EuF_3_ NPs occurred, which can be explained by the theory of hard and soft acids and bases (HSAB)^32^. The so called “hard” acids and “hard” bases prefer to bond with each other, and so do the “soft” acids and “soft” bases. In our reaction system, F^−^ ion as a base is harder than O^2−^ and similarly H^+^ as an acid is harder than Eu^3+^. Therefore, the Eu-O bond was broken under the attack of H^+^, which results in the semi-dissociation of EuOF NPs and release of non-bonded Eu^3+^ and F^−^ ions. Then Eu^3+^ preferentially bonds with F^−^ rather than O^2−^ by forming tiny EuF_3_ NPs^33^. The chemical equation can be expressed as:

1 $${\rm{EuOF}}+{\rm{HCl}}\to {{\rm{EuF}}}_{3}+{{\rm{EuCl}}}_{3}+{{\rm{H}}}_{2}{\rm{O}}$$

Moreover, disappearance of FT-IR peaks at ~2923, 2852, 1560 and 1466 cm^−1^ clearly indicates debond of OA^−^ ligands from surface Eu^3+^. The conversion of EuOF NPs to EuF_3_ NPs completes at reaction time of 5 min (Supplementary Fig. 5). This is also consistent with the change of solution from opaque to transparent at 5 min (Supplementary Fig. 2), which is due to the improved solubility of EuF_3_ NPs through solvent molecule (H_2_O or C_2_H_5_OH) coordination, as proved by the broad hydroxyl peak at ~3400 cm^−1^ (ref. ^34^) (Fig. 2i).

Stage 2: Initiation of the assembly. Presence of OA^-^ improves solubility and mitigates collision probability of NPs in solution, which allows long-term storage of LnOF NPs precursors without worrying about aggregation (Supplementary Fig. 1). However, surface ions become exposed once OA^−^ is removed, causing sharply risen surface energy of EuF_3_ NPs^29^ and initiates the assembly (Fig. 2c).

Stage 3: Growth of EuF_3_ NCs. The size of EuF_3_ NCs grew from 3.1 to 21.8, 29.2, 35.7, 38.4, and 40.1 nm at the assembly time of 15, 30, 60, 120, 240, and 360 min, respectively (Fig. 2c–h, j and Supplementary Fig. 6). Parallelly, the FT-IR peaks at 2852 and 2923 cm^−1^ corresponding to –CH_3_ stretching vibration intensified from almost nil at 5 min to that of 30 min and further at 360 min (Fig. 2i). This is reasonable because trace amount of OA^−^ on the surface of individual EuF_3_ NP can’t produce sufficient FT-IR signal at stages 1 and 2. More and more ligands are accumulated (Fig. 1c, d) when NCs grow larger as assembly progresses, resulting in stronger signals. This also well agrees with the solubility change in ethanol solution from transparent to opaque (Supplementary Fig. 2b, c). Thus, the intensity variation of FT-IR peaks at 2852 and 2923 cm^−1^ (Fig. 2i) could qualitatively unveil the structural details of NCs formed at different assembly stages.

### Driving force for EuF_3_ NCs assembly

As previously reported^35^, multiple interactions usually work together to make for the final structure of assemblies. In our case, removal of surface ligands from NPs not only exposes a large number of uncoordinated Eu^3+^, but also leads to increased surface energy. In this situation, unstable EuF_3_ NPs tend to contact and attach with each other to decrease the total energy through interparticle interactions, and eventually assemble into NCs. The FT-IR peak at 3400 cm^−1^ implies the existence of hydrogen bond among solvent molecules, while the partially fused crystal lattices (Fig. 1d) suggest the formation of Eu-F chemical bond among EuF_3_ NPs, both of which are the main interactions responsible for the assembly of EuF_3_ NCs through a random attachment process (Fig. 1c, d). Moreover, as widely reported^36,37^, dipole-induced interaction is a strong force at the nanoscale to drive self-assembly, which usually occurs in NPs with asymmetric crystal lattice. Considering the trigonal crystal structure of EuF_3_ NPs with relatively low symmetry among the seven crystal systems and the big difference in electronegativity between F^−^ and Eu^3+^, there might exist electric dipole moment originated from the polarity of crystal lattice of EuF_3_ NPs, which may generate dipole-dipole interactions between EuF_3_ NPs and act as a driving force for the self-assembly process.

More intriguingly, although the as-assembled EuF_3_ NCs are polycrystalline, they are readily to be converted into single crystals when annealed at temperatures as low as 50 °C (Supplementary Fig. 7). This principle might be instructive to the synthesis of nanocrystals or nanoalloys that are not possible at nearly room temperature. Moreover, NCs of NdF_3_, SmF_3_, and GdF_3_ were also successfully constructed by following the same strategy (Supplementary Figs. 8 and 9).

### Working principle of Eu^3+^ as luminescent probe

**Fig. 3:**
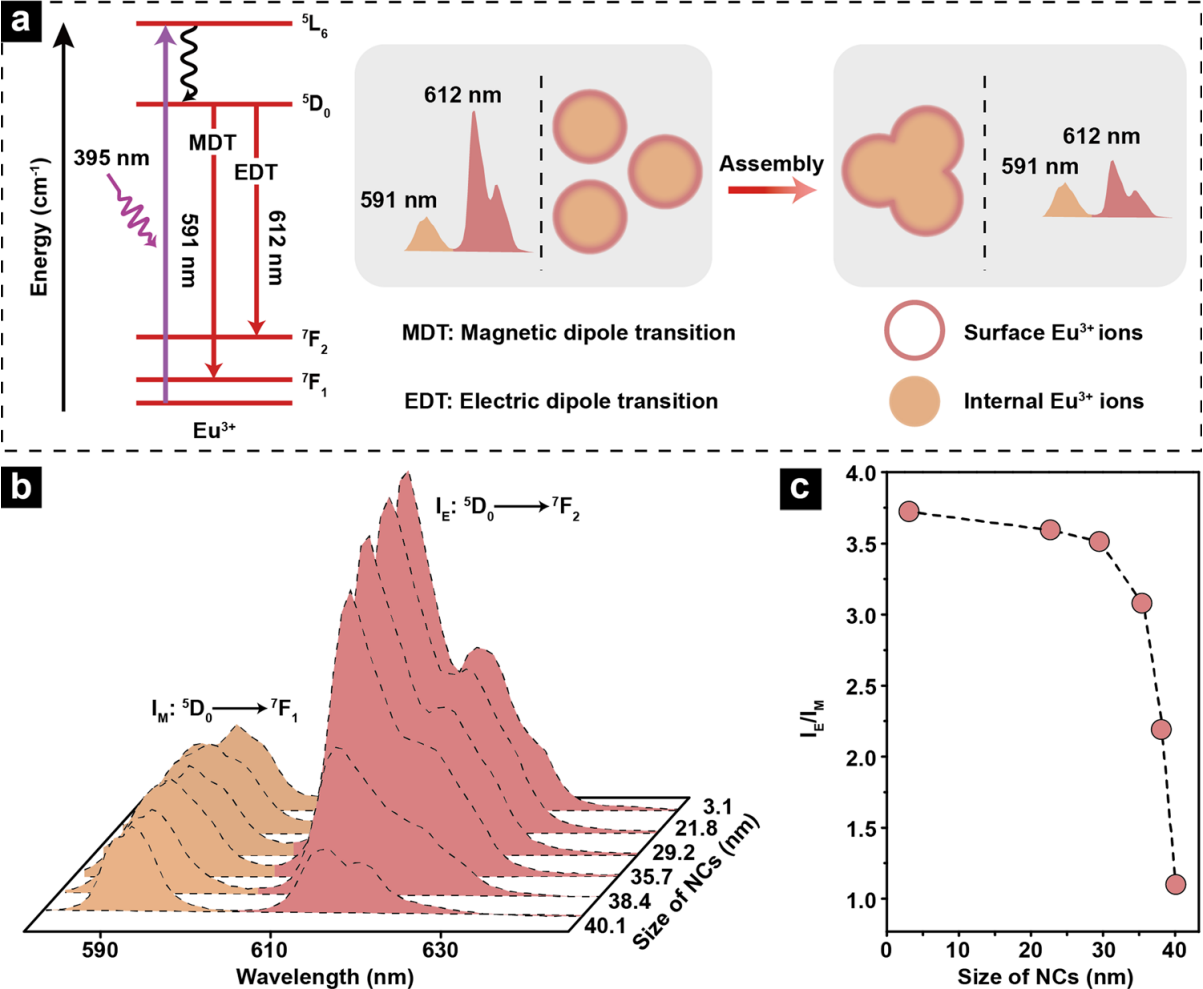
Working principle of Eu^3+^ used as luminescent probe. **a** Illustration of different electron transition pathways in Eu^3+^ and intensity evolutions of electric-dipole (612 nm) and magnetic-dipole (591 nm) transitions during NCs growth. **b** Evolution of PL spectra and **c** I_E_/I_M_ value of Eu^3+^ in NCs with different sizes. 395 nm light was used as excitation source.

As two major photoluminescence (PL) emissions in Eu^3+^, the intensity at 612 nm corresponding to the ^5^D_0_→^7^F_2_ electric dipole transition (I_E_) is highly sensitive to the local site symmetry while that of the ^5^D_0_→^7^F_1_ magnetic dipole transition (I_M_) at 591 nm is not^38^ (Fig. 3a). Considering that local site symmetry of luminescent ions, here means Eu^3+^, usually links with surface coordination environment, evolution of the value of I_E_/I_M_ may be used as a luminescent probe to track the changes of the proportion of surface Eu^3+^ in different assemblies (Fig. 3a, b and Supplementary Table 1). Indeed, when excited at the characteristic working wavelength of Eu^3+^ (395 nm), the calculated I_E_/I_M_ decreases gradually from 3.80 in EuF_3_ NPs to 1.14 in EuF_3_ NCs, indicating that the amount of surface Eu^3+^ keeps reducing with the size increase of NCs (Fig. 3b, c)^39^. This well-agrees with the mergence of NPs when forming NCs, which causes reduced surface area (Figs. 1d and 3a).

It is worth pointing out that the gradually decrescent full-width at half maximum (FWHM) at 591 nm is due to the increased site symmetry of Eu^3+^ during NCs growth^38^. Generally, the crystallographic site symmetry significantly affects the spectral line-width of Eu^3+^, and a minor structural distortion may alter the crystal field symmetry of Eu^3+^, which will be reflected by the gradual change of FWHM from 12.1 to 7.2 nm as the NCs grow from 3.1 to 40.1 nm^40,41^.

### Construction of arbitrarily designed NCs

**Fig. 4:**
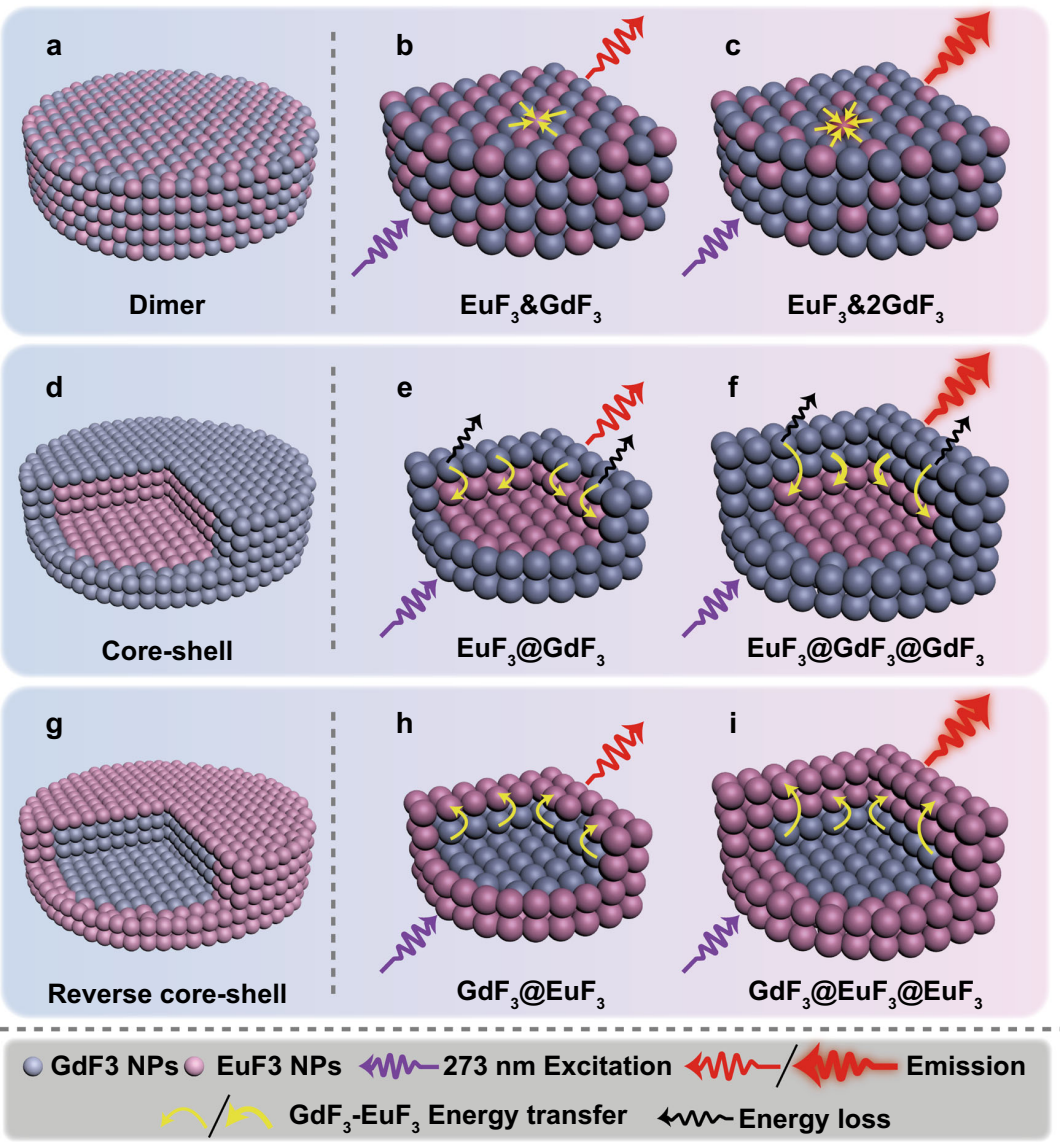
Schematic illustration of the structures and energy transfer pathways of different NCs. The structures of **a** dimeric, **b** EuF_3_&GdF_3_, and **c** EuF_3_&2GdF_3_ NCs. **d** Core-shelled, **e** EuF_3_@GdF_3_, and **f** EuF_3_@GdF_3_@GdF_3_ NCs. **g** Reversely core-shelled, **h** GdF_3_@EuF_3_, and **i** GdF_3_@EuF_3_@EuF_3_ NCs, as well as their respective energy transfer pathways. **b**, **c**, **e**, **f** and **h**, **i** illustrate the energy transfer details in differently structured NCs using 273 nm light to excite Gd^3+^, which transfers energy to Eu^3+^ (Supplementary Fig. 11). Note: for simplicity, energy migrations between Gd^3+^–Gd^3+^ and Eu^3+^–Eu^3+^ were not shown.

Clear understanding of the NC formation mechanisms, particularly the information extracted from luminescent probe has inspired us to further fabricate more complicated NCs and explore their structural relationships with the evolution of I_E_/I_M_. Instead of the-above uninterrupted NC assembly (Fig. 2), the conversion of LnOF NPs to and the following assembly of LnF_3_ NPs were purposely separated, which offers more freedom to grow NCs with widely varied structures and pre-designed chemical compositions. For example, dimeric NCs of EuF_3_&GdF_3_ and EuF_3_&2GdF_3_ could be reliably constructed by simply mixing NPs of EuF_3_ and GdF_3_ that were pre-converted from EuOF and GdOF NPs via a H^+^-induced clickable reaction, at molar ratios of 1:1 and 1:2, respectively (Fig. 4a–c). Similarly, core-shelled NCs of EuF_3_@GdF_3_ and EuF_3_@GdF_3_@GdF_3_ could also be fabricated by controlling the number of GdF_3_ shells grown on pre-formed core of EuF_3_ NCs (Fig. 4d–f) and alternatively, the reversely core-shelled NCs of GdF_3_@EuF_3_ and GdF_3_@EuF_3_@EuF_3_ were further grown by reversing the assembly sequence of respective components (Fig. 4g–i).

### Luminescent probes for tracking of structural variations and energy transfer pathways

Compared with previous assembly mechanism studies where HRTEM, XRD, and synchrotron characterizations were heavily relied^42,43^, information provided by luminescent probes can be in situ, dynamic, precise, and particularly sensitive to its surrounding environment. Indeed, as depicted in Fig. 4, different Gd^3+^–Eu^3+^ energy transfer modes were clearly distinguished in their respective PL spectral evolution, which could be utilized to track the distribution variation of surface Eu^3+^ on NCs with different structures.

**Fig. 5:**
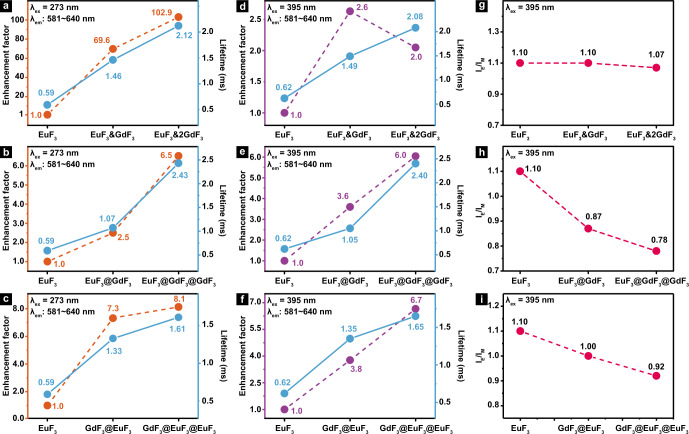
The PL evolution in different-structured NCs. **a**–**c** Variation of luminescence enhancement factor (orange lines) and lifetime (baby blue lines) of Eu^3+^ in different NCs under 273 nm light excitation, through Gd^3+^–Eu^3+^ energy transfer. **d**–**f** Luminescence enhancement factor (purple lines) and lifetime (baby blue lines) of Eu^3+^ in different NCs under 395 nm light excitation. **g**–**i** Normalized I_E_/I_M_ value of Eu^3+^ in different NCs.

For pure EuF_3_ NCs, there is only very weak PL because Eu^3+^ has almost no absorption at 273 nm (Supplementary Fig. 10a, b). However, a 70-fold emission enhancement (Fig. 5a) was seen in EuF_3_&GdF_3_ NCs, which is rational because 273 nm is the characteristic working wavelength of Gd^3+^ and more energy is transferred to Eu^3+^ in the co-assembled NCs (Fig. 4b and Supplementary Fig. 11). Subsequently, a 103-fold PL enhancement was obtained in EuF_3_&2GdF_3_ NCs due to further increased energy transfer from Gd^3+^ to Eu^3+^ (Fig. 4c). Consistently, the characteristic absorption peak of Gd^3+^ at 273 nm appeared in EuF_3_&GdF_3_ and became stronger in EuF_3_&2GdF_3_, proving the successful assembly of GdF_3_ NPs at increased amount (Supplementary Fig. 10a). More excitingly, such PL evolution also agrees with the gradually intensified optical images of respective samples (Supplementary Fig. 12a) under 273 nm light excitation where EuF_3_&2GdF_3_ is the brightest. Correspondingly, the lifetime of Eu^3+^ (Fig. 5a and Supplementary Fig. 13a) shows similar trend of increase from EuF_3_ NPs (0.59 ms) to EuF_3_&GdF_3_ (1.46 ms) as that from EuF_3_&GdF_3_ (1.46 ms) to EuF_3_&2GdF_3_ (2.12 ms). As a stark contrast, neither emission intensity nor lifetime change was observed in Eu^3+^ under 273 nm light excitation (Supplementary Fig. 14) when solutions containing pre-formed NCs of GdF_3_ and EuF_3_ were physically mixed at the atomic ratios of 2:1, 1:1, and 0:1, respectively. This proves straightforwardly that Gd^3+^–Eu^3+^ energy transfer can hardly occur if without uniform co-assembly between EuF_3_ and GdF_3_ NPs (Fig. 4a–c).

Similarly, Gd^3+^ in GdF_3_ shell keeps transferring energy to internal Eu^3+^, resulting in enhanced PL in EuF_3_@GdF_3_ NCs (Fig. 5b and Supplementary Fig. 10c, d). However, it is reasonable to see only 2.5-fold enhancement because there exists partial energy loss from surface Gd^3+^ on GdF_3_ shell (Fig. 4e). Indeed, an enhancement of 6.5 folds is seen in EuF_3_@GdF_3_@GdF_3_ NCs, which is due to: 1) increased amount of Gd^3+^ that transfers more energy to Eu^3+^ and meanwhile, 2) decreased amount of Gd^3+^ energy loss due to the growth of the second GdF_3_ shell (Fig. 4f). Accordingly, there is a larger Eu^3+^ lifetime increase (Fig. 5b and Supplementary Fig. 13b) after growth of the second GdF_3_ shell (from 1.07 to 2.43 ms) compared with that of the first GdF_3_ shell (from 0.59 to 1.07 ms).

Interestingly, more enhanced (7.3-fold) PL is seen in GdF_3_@EuF_3_ NCs than that of 2.5-fold in EuF_3_@GdF_3_ NCs (Fig. 5b, c and Supplementary Fig. 10e, f). This is due to more efficient energy transfer from internal Gd^3+^ to exterior Eu^3+^ where energy loss in Fig. 4e was largely impressed in Fig. 4h. However, the reason that only 8.1-fold PL enhancement is gained after growth of a second EuF_3_ shell is because the amount of energy transferred from Gd^3+^ is fixed so that the second layer of Eu^3+^ can only absorb the extra energy that passes through the first Eu^3+^ shell (Fig. 4i). Rationally, the lifetime increase of Eu^3+^ (Fig. 5c and Supplementary Fig. 13c) is not as much after growth of the second EuF_3_ shell (from 1.33 to 1.61 ms) compared with that of the first (from 0.59 to 1.33 ms). It is worth emphasizing that the calculated quantum yields^39,44^ of EuF_3_ (Supplementary Equation (1) and Supplementary Table 2) are consistent with above-discussed PL intensity evolution, and variation of the calculated GdF_3_-EuF_3_ energy transfer rate^45–48^ in different NCs based on simplified models (Supplementary Fig. 15) provides further support to our schematic illustrations in Fig. 4.

To dig out more structural information from another point of view, the PL evolution in different NCs was investigated by directly exciting Eu^3+^ at 395 nm (Supplementary Fig. 16). For easy comparison, the PL intensity in pure EuF_3_ NCs was set at 1.0 and the calculated enhancement factors in different NCs were shown in Fig. 5d–f. It is reasonable to see enhanced PL in EuF_3_&GdF_3_ and EuF_3_&2GdF_3_ because there exists a Eu^3+^–Eu^3+^ energy migration loop (Supplementary Fig. 16a and Supplementary Fig. 17a–c) in pure EuF_3_ NCs^49^ that deteriorates the emission while insertion of co-assembled GdF_3_ NPs breaks the loop and leads to enhanced PL in EuF_3_&GdF_3_. It is also reasonable to see a 2.6- and 2.0-fold enhancement because the unit number of Eu^3+^ is diluted 2 and 3 times in EuF_3_&GdF_3_ and EuF_3_&2GdF_3_ (Fig. 5d), respectively. In another word, there shall be a 5.2- and 6-fold absolute PL enhancement in EuF_3_&GdF_3_ and EuF_3_&2GdF_3_, respectively. This phenomenon is quite instructive for materials design towards maximized PL intensity while minimizing the amount of activators used. Parallelly, the lifetime of Eu^3+^ also increases from 0.62 to 1.49, and 2.08 ms (Supplementary Fig. 16b).

Growth of a GdF_3_ shell suppresses the energy loss of surface Eu^3+^ similar to that of Gd^3+^ in Fig. 4e and results in 3.6-fold PL enhancement in EuF_3_@GdF_3_, while the 6.0-fold PL enhancement in EuF_3_@GdF_3_@GdF_3_ suggests that the second shell has even better energy loss suppression (Supplementary Fig. 16c). This is also consistent with the lifetime increase of Eu^3+^ from 0.62 to 1.05, and 2.40 ms (Supplementary Fig. 16d). Alternatively, growth of a EuF_3_ shell around pre-formed core of GdF_3_ NCs results in larger inter-particle distance, which generates similar (3.8-fold) effect as that in EuF_3_&GdF_3_ (Supplementary Fig. 17a–c), that is, the broken energy migration loop and enhanced PL (Supplementary Fig. 17a, d and e). Growth of the second EuF_3_ shell not only suppresses the energy loss of Eu^3+^ in the first EuF_3_ shell, the increased amount of Eu^3+^ generates extra PL so that a total enhancement of 6.7-fold is obtained in GdF_3_@EuF_3_@EuF_3_ NCs (Supplementary Fig. 16e). Accordingly, the lifetime of Eu^3+^ increases from 0.62 to 1.35 and 1.65 ms (Fig. 5f and Supplementary Fig. 16f).

Moreover, based on the principle discussed in Fig. 3a, evolution of I_E_/I_M_ value directly links with the variation of surface Eu^3+^ proportion in different NCs where tiny structural difference may be precisely probed. Indeed, for EuF_3_&GdF_3_ NCs with different EuF_3_/GdF_3_ molar ratios, the I_E_/I_M_ remains almost constant (Fig. 5g), which is reasonable because EuF_3_ and GdF_3_ NPs are mixed uniformly during co-assembly process. So, no matter what the EuF_3_/GdF_3_ molar ratio is, the proportion of surface Eu^3+^ compared to internal Eu^3+^ remains unchanged, which agrees exactly with the proposed structure in Fig. 4a.

Similarly, compared with pure EuF_3_ NCs, growth of external GdF_3_ shell sharply reduces the proportion of surface Eu^3+^, so I_E_/I_M_ value declined rapidly from 1.10 in EuF_3_ NCs to 0.87 in EuF_3_@GdF_3_ and further to 0.78 in EuF_3_@GdF_3_@GdF_3_ (Fig. 5h). The I_E_/I_M_ value of Eu^3+^ also declined (Fig. 5i) from 1.10 in EuF_3_ NCs to 1.00 in GdF_3_@EuF_3_ NCs and 0.92 in GdF_3_@EuF_3_@EuF_3_ NCs because enlarged NCs shows lower proportion of surface Eu^3+^.

**Fig. 6:**
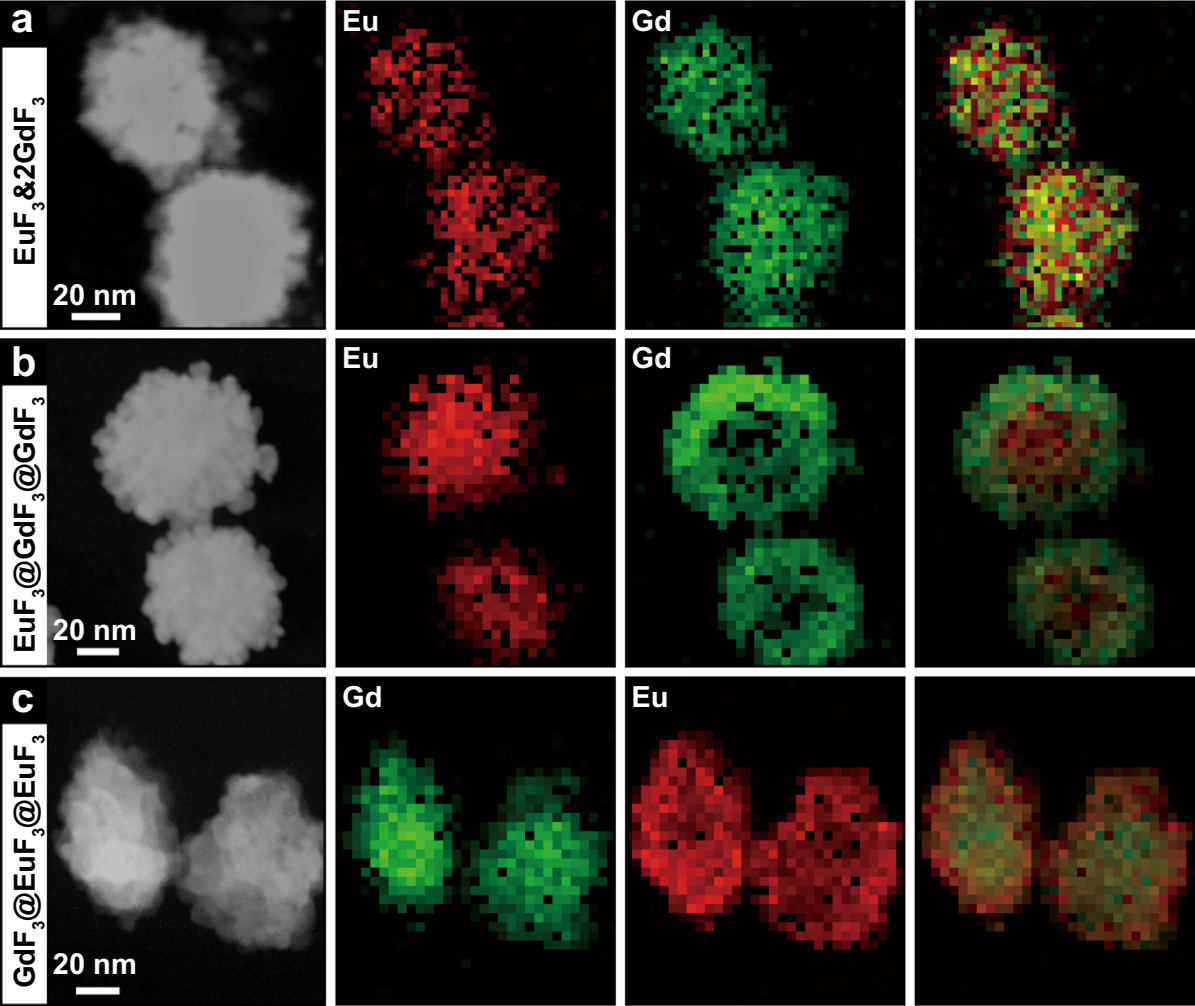
Structural details of different NCs. High-angle annular dark-field scanning TEM images and corresponding energy-dispersive X-ray elemental mapping results of **a** EuF_3_&2GdF_3_ dimeric, **b** EuF_3_@GdF_3_@GdF_3_ core-shelled, and **c** GdF_3_@EuF_3_@EuF_3_ reversely core-shelled NCs.

Although TEM images only show morphological and dimensional differences of the above NCs (Supplementary Figs. 18, 19 and 20), the elemental mapping results provide unambiguous evidence for the structural differences of the purposely fabricated NCs illustrated in Fig. 4. For example, uniform distribution of Eu and Gd signals in the structure of EuF_3_&2GdF_3_ (Fig. 6a) suggests the complete co-assembly of EuF_3_ and GdF_3_ NPs, as depicted in Fig. 4c. Similarly, the opposite Eu and Gd signal distribution in Fig. 6b and c perfectly matches the core-shelled structure of EuF_3_@GdF_3_@GdF_3_ (Fig. 4d) and reversely core-shelled structure of GdF_3_@EuF_3_@EuF_3_ (Fig. 4g), respectively. These results are also highly consistent with both our principle designs and the structural details extracted from PL signals generated by the embedded dual luminescent probes of Gd^3+^ and Eu^3+^.

## Discussion

We have developed a clickable assembly strategy that allows facile construction of arbitrary NCs from individual LnF_3_ NPs. As a complement to widely used one-step assembly, this two-step method enables fabrication of more versatile nanoarchitectures such as clusters, dimers, core-shells, and more importantly with multiple components and tunable functionalities without worrying about aggregation of precursors. It is worth emphasizing that the in-situ, sensitive, and dynamic PL signal generated by the embedded luminescent probes offers unparalleled superiority in precisely tracking not only tiny structural variations of but also energy transfer pathways in complex nanoarchitectures. Considering the large family of OA-stabilized NPs and principally other systems, this work has paved a potential avenue to the fabrication of widely diversified NCs with promising functionalities in the areas of catalysis, optoelectronics, energy conversion/harvesting, nanoalloys, bioimaging, as well as theranostics.

## Methods

### Materials

Oxides of Ln_2_O_3_ (99.99%, Ln = Eu, Gd, Sm, and Nd) were purchased from Beijing HWRK Chem. Co. LTD. Oleic acid (OA, 90%) and oleylamine (OM, 70%) were purchased from Sigma-Aldrich. Trifluoroacetic acid (TFA) and hydrochloric acid (HCl) was purchased from Shanghai Lingfeng Chemical Company, China. All the chemicals were used as received without further purification.

### Preparation of Ln(CF_3_COO)_3_

In a typical synthesis, lanthanide oxide was added into the aqueous solution containing slightly excessive trichloroacetic acid with continuous stirring, which was then kept refluxing so as to form an optically transparent solution. The resulting solution was filtered to remove the insoluble materials (if any), and the following solution was dried in an oven at 85 °C for 24 h to obtain Ln(CF_3_COO)_3_.

### Synthesis of LnOF NPs

Typical procedure: a given amount of Ln(CF_3_COO)_3_ (2 mmol) was added into the mixture of OA (20 mmol) and OM (20 mmol) in a three-necked flask (100 mL) at room temperature. The slurry was heated to 100 °C with vigorous magnetic stirring under vacuum for 10 min in a temperature-controlled electromantle to remove water and oxygen, thereby forming an optically transparent solution, which was then ramped to 310 °C at a heating rate of 10 °C/min and maintained for 2 h under N_2_ atmosphere. When cooling down to room temperature, the NPs were precipitated by adding excessive absolute ethanol into the reaction solution, followed by washing with ethanol for three times. Then the as-prepared NPs were dispersed into cyclohexane for further use.

### Self-assembly of LnOF NPs into LnF_3_ NCs

The NCs assembled from LnOF NPs were obtained through an acid treatment process. Typically, a certain amount of LnOF NPs were added into 4 mL ethanol, which was then poured into a 20 mL beaker and stirred for 5 min. 200 μL diluted hydrochloric acid solution (1 M) was added into the above solution and stirred at around 1000 r/min. After 6 h, the assembled NCs were generated in the solution, which was centrifuged at a speed of 8000 r/min for 4 min to obtain the NC samples, and then washed twice with ethanol under this condition to obtain the final NC samples.

### Self-assembly of EuF_3_&GdF_3_ dimeric NC

Take the EuF_3_&GdF_3_ dimeric NC (molar ratio of EuF_3_:GdF_3_ = 1:1) as an example: typically, a certain amount of EuOF NPs and GdOF NPs were transferred into 4 mL ethanol solution, respectively, which was poured into 20 mL beaker, and stirred for 5 min for complete dispersion. Then, 200 μL diluted hydrochloric acid solution (1 M) was added respectively into the above EuOF and GdOF solutions and kept stirring at around 500 r/min. After 10 min, the EuOF NPs and GdOF NPs totally converted into EuF_3_ NPs and GdF_3_ NPs, respectively. Then, solutions of EuF_3_ NPs and GdF_3_ NPs were mixed together into 20 mL beaker, and stirred at 1000 r/min. After 6 h, the above solution was centrifuged at a speed of 8000 r/min for 4 min to obtain the EuF_3_&GdF_3_ dimeric NCs, which were then washed with ethanol twice under this condition to obtain the final products.

### Self-assembly of core-shelled NCs

Take the EuF_3_@GdF_3_ core-shelled NCs (molar ratio of EuF_3_:GdF_3_ = 1:1) as an example: typically, a certain amount of EuOF NPs was transferred into 4 mL ethanol solution, poured into 20 mL beaker, and stirred for 5 min. 200 μL diluted hydrochloric acid solution (1 M) was then added into the above solution and kept stirring for 6 h, until the assembled EuF_3_ NCs were formed. The such-obtained EuF_3_ NCs were further used as core for EuF_3_@GdF_3_ core-shell structured NCs growth. Meanwhile, a certain amount of GdOF NPs was transferred into 4 mL ethanol solution, poured into 20 mL beaker, and stirred for 5 min. Subsequently, 200 μL diluted hydrochloric acid solution (1 M) was added into the above GdOF solution and kept stirring for 10 min, until GdOF NPs totally converted into GdF_3_ NPs. Then, solutions containing GdF_3_ NPs and EuF_3_ NCs were mixed together at certain ratio, and stirred for 6 h. The solution was centrifuged at 8000 r/min for 4 min to obtain the EuF_3_@GdF_3_ core-shelled NCs, which were then washed twice with ethanol for future analysis.

### Characterization

Powder X-ray diffraction (XRD) analysis was carried out on a Rigaku Smartlab (3 kW) X-ray diffractometer (Rigaku Corporation, Japan) using Cu Kα radiation (*λ* = 1.5406 Å) and the measurement was performed at ambient temperature in the range of 2θ = 10-60 degree with 0.02 degree/step. Transmission electron microscope (TEM) images were recorded on a JEOL-1400 Plus (JEOL Ltd, Japan) at an acceleration voltage of 120 kV. High-resolution TEM (HRTEM) images and Energy-dispersive X-ray (EDX) spectra were obtained on a Tecnai G2 F20 TEM (FEI, America) at an acceleration voltage of 200 kV.

Fourier transform infrared (FT-IR) spectra were collected on a Nicolet 380 FT-IR spectrometer (Nicolet, America). Photoluminescence (PL) emission spectra were recorded on an Edinburg FLS1000 Photoemission Spectrometer (Edinburgh Instruments, UK). All luminescent characterizations including PL emission spectra, excitation spectra, and luminescence decay curves were collected at 25 °C. Excitation and emission spectra were measured by using a 450 W continuous xenon arc lamp for sample excitation, and detailed parameters are shown in Supplementary Table 1. Luminescence decay curves were measured by using a compact 60 W xenon flash lamp (μF2) as excitation source with the frequency of 40 Hz.

## Supplementary information

Supplementary Information

## Data Availability

All data supporting the findings of this study are available within the paper and its supplementary information files.
