# Supplementary material for: A luminescent view of the clickable assembly of LnF3 nanoclusters
Source: Nat Commun. 2021 May 19;12:2948. doi: 10.1038/s41467-021-23176-y (PMC8136472; doi:10.1038/s41467-021-23176-y)
Supplement: Supplementary file 1 — Supplementary Information [file 41467_2021_23176_MOESM1_ESM.pdf]

SUPPLEMENTARY INFORMATION

A Luminescent View of the Clickable Assembly of LnF_3 Nanoclusters

Zhou et al.

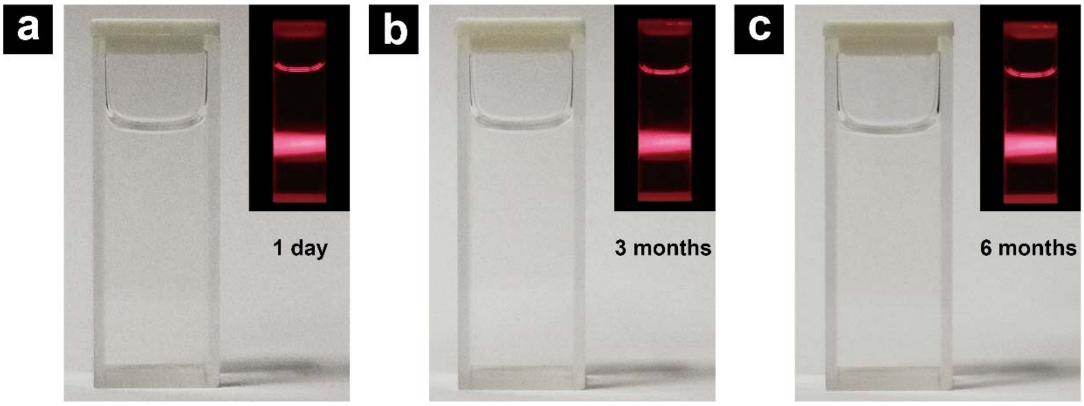

Supplementary Fig. 1 Stability test of EuOF NPs. Digital photos and corresponding luminescent photos (insets) of EuOF NPs solutions stored for **a** 1 day, **b** 3 months, and **c** 6 months. These results suggest that the precursor solution of EuOF NPs protected by OA ligands can be stored for very long time without worrying about aggregation. 395 nm light that is the working wavelength of Eu^{3+} was used as excitation source.

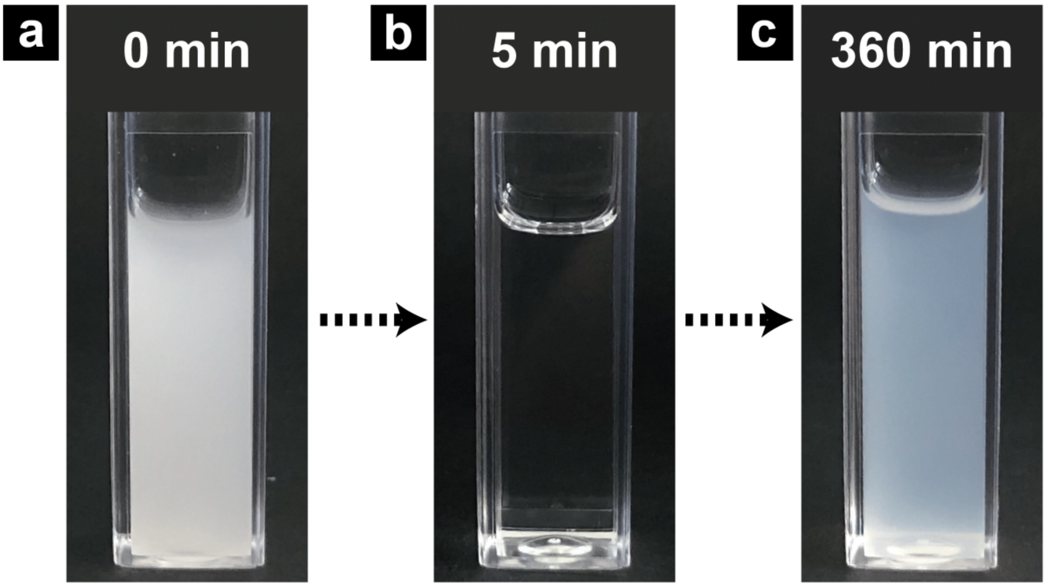

Supplementary Fig. 2 Solubility change of the products at different self-assembly stages. Digital photos of ethanol solutions of **a** EuOF NPs, **b** EuF_3 NPs, and **c** EuF_3 NCs. Within just 5 mins, the ethanol solution of EuOF NPs gradually changed from opaque to transparent after addition of 200 μL HCl (1 M), suggesting the removal of OA^- ligands and conversion of EuOF NPs to EuF_3 NPs. The successive assembly of EuF_3 NPs leads to accumulation of OA^- ligands on EuF_3 NCs, which decreases the solubility in ethanol and results in opaque again.

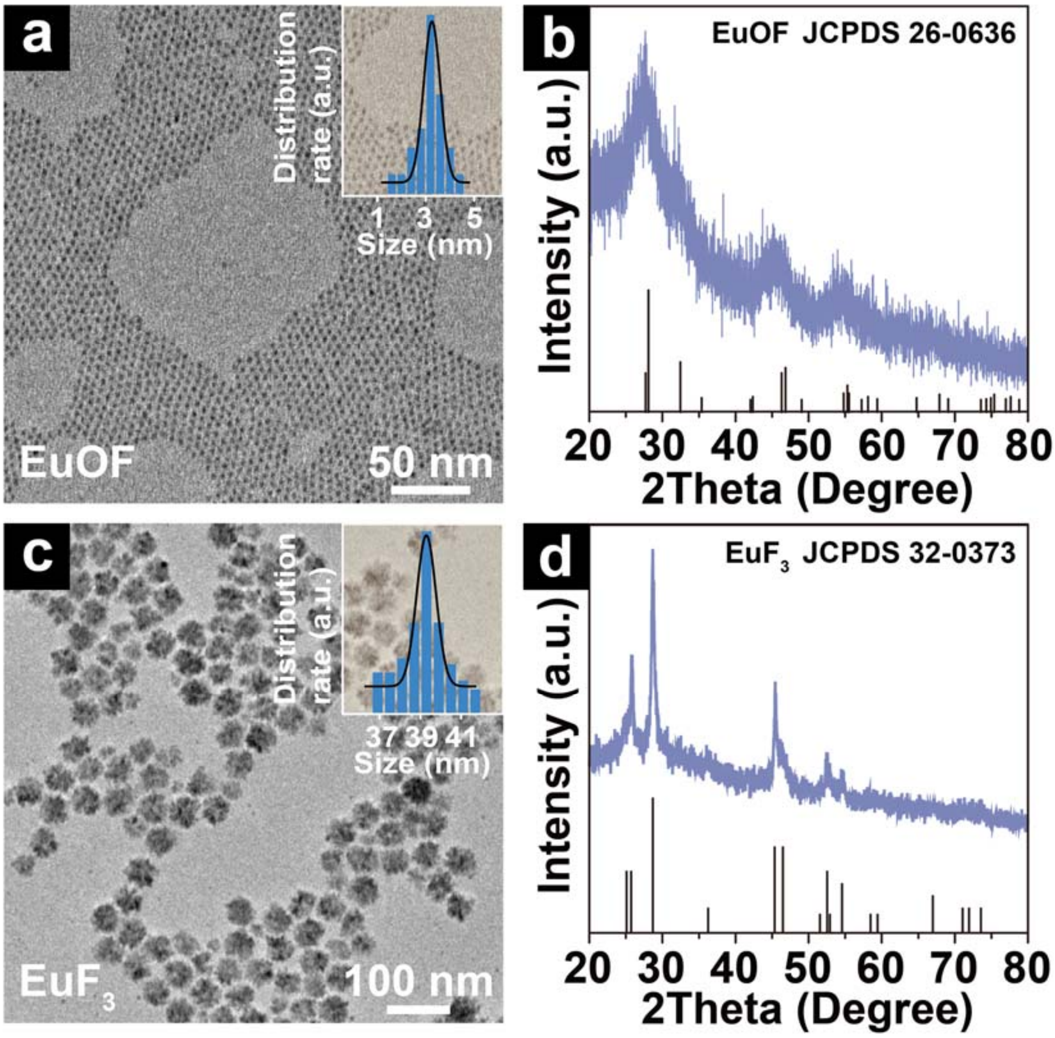

Supplementary Fig. 3 Characterization of EuOF NPs and EuF_3 NCs. TEM images of **a** EuOF NPs and **c** EuF_3 NCs. XRD patterns of **b** EuOF NPs and **d** EuF_3 NCs. Insets in **a** and **c** show the size distributions of EuOF NPs and EuF_3 NCs, respectively.

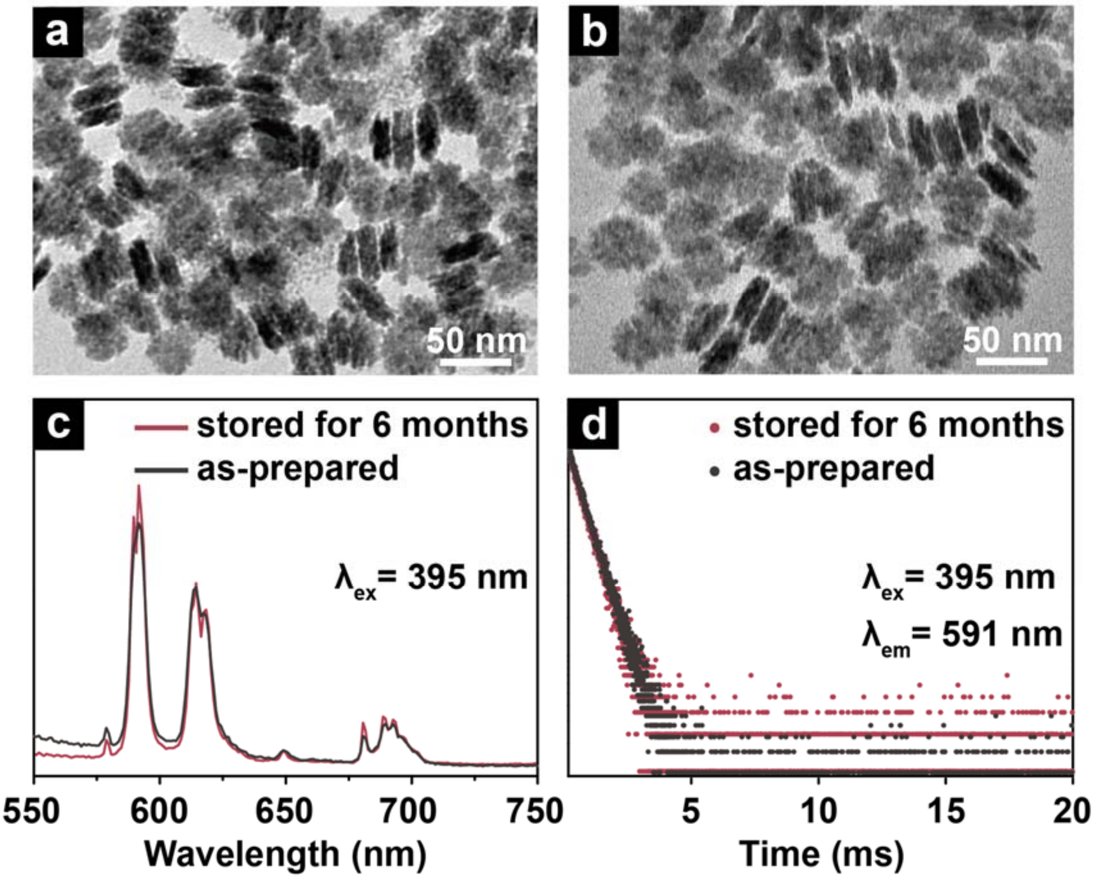

Supplementary Fig. 4 Stability characterization of as-synthesized NCs. The self-assembled EuF_3 NCs were all dispersed in 2 mL ethanol and kept in fresh-keeping layer (5 °C) of the refrigerator. TEM images of **a** as-prepared EuF_3 NCs and **b** the EuF_3 NCs kept in ethanol solution at 5 °C for 6 months show no difference, and their **c** PL spectra and **d** luminescence decay curves remain almost identical, suggesting that EuF_3 NCs are highly stable. PL spectra were collected using the 395 nm light as excitation source.

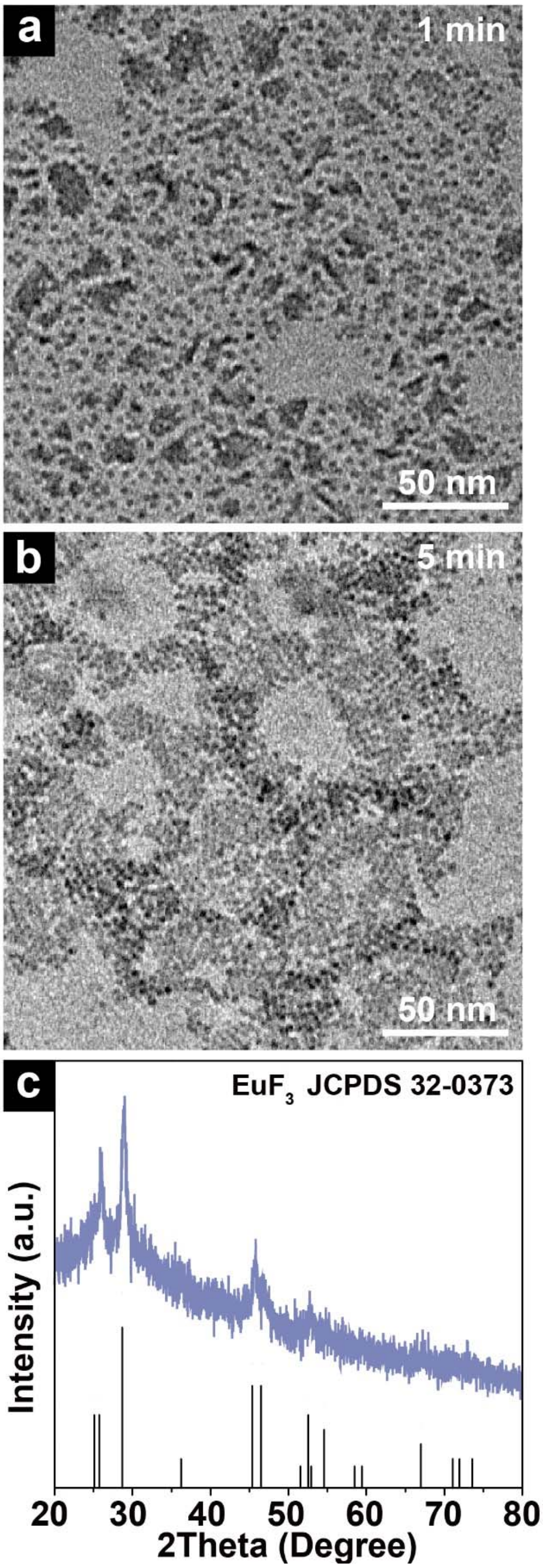

Supplementary Fig. 5 Observation of the generation of EuF_3 NPs. TEM images of EuF_3 NPs obtained at the assembly time of **a** 1 min and **b** 5 min. **c** XRD patterns of EuF_3 NPs obtained at assembly time of 5 min.

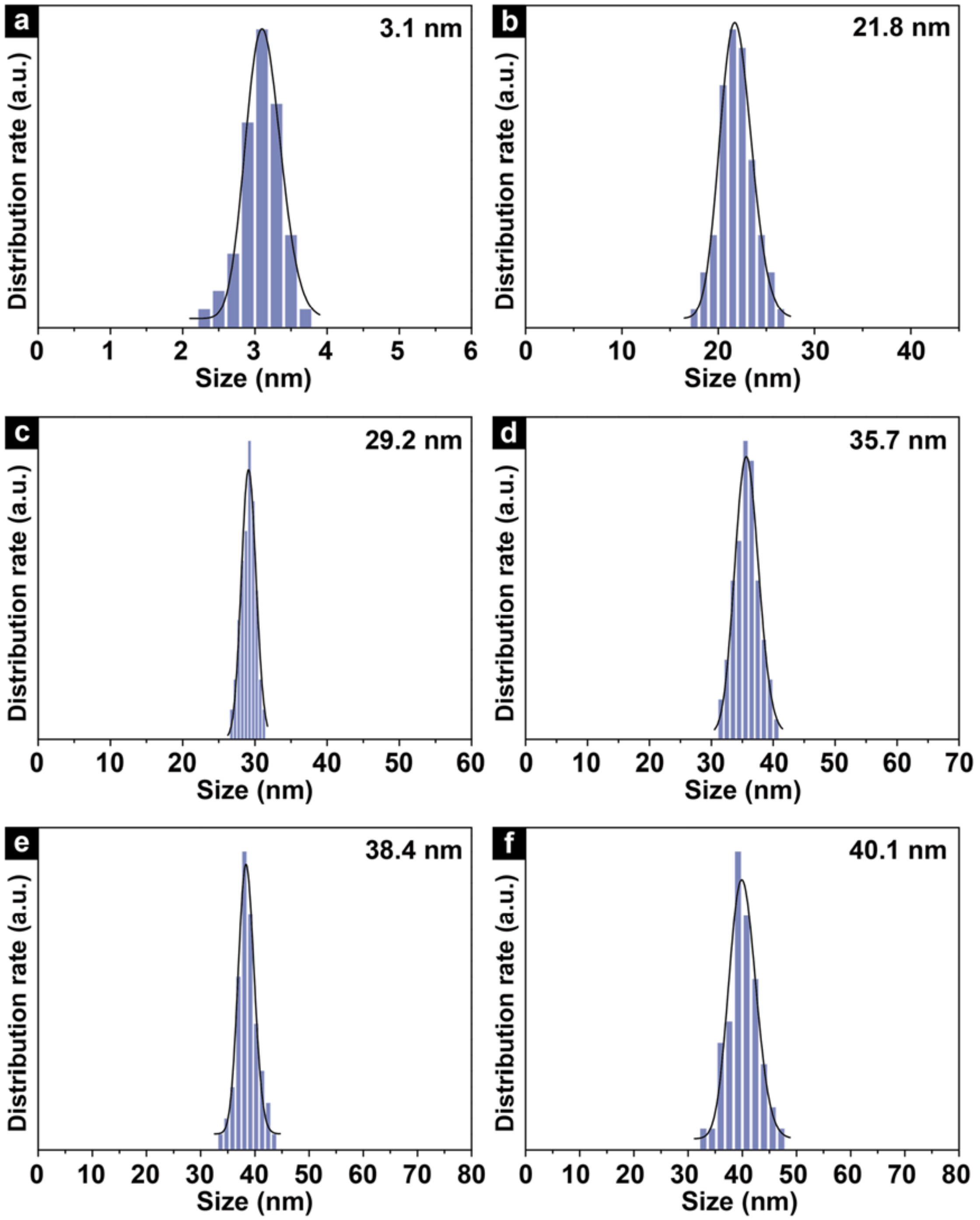

Supplementary Fig. 6 Time-dependent size evolution of EuF_3 NCs. Statistics of time-dependent size distributions of NCs at the assembly time of **a** 15, **b** 30, **c** 60, **d** 120, **e** 240, and **f** 360 min, respectively (the size distributions were fitted by log-normal function).

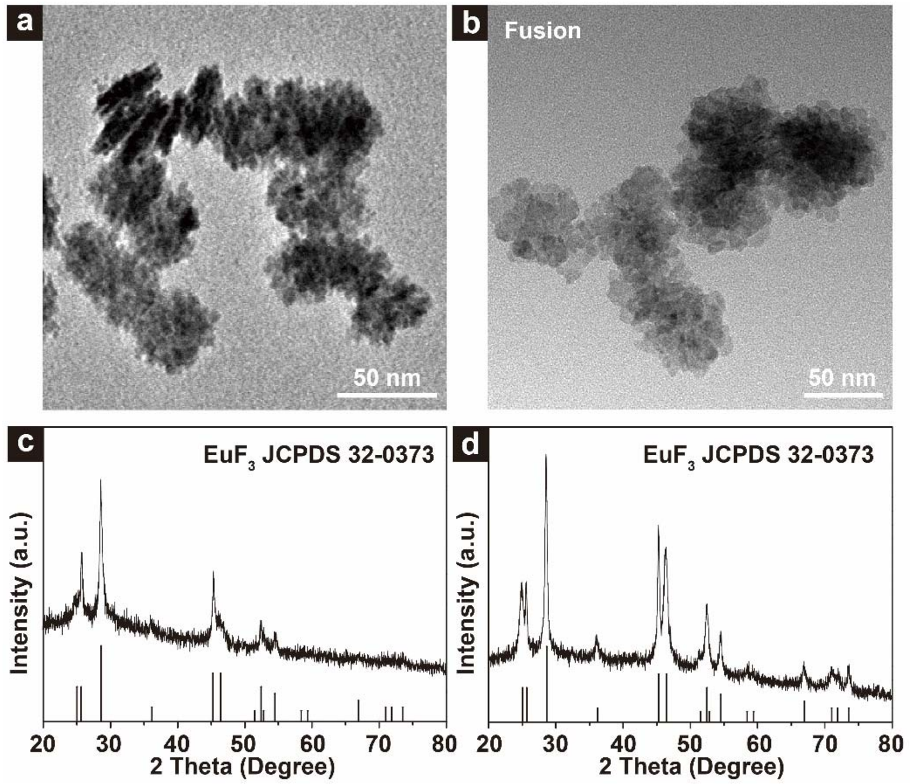

Supplementary Fig. 7 Characterization of EuF_3 NCs before and after annealing. **a** TEM image and **c** XRD patterns of EuF_3 NCs assembled at room temperature. **b** TEM image and **d** XRD patterns of EuF_3 NCs obtained after being annealed at 50 °C for 6 hours.

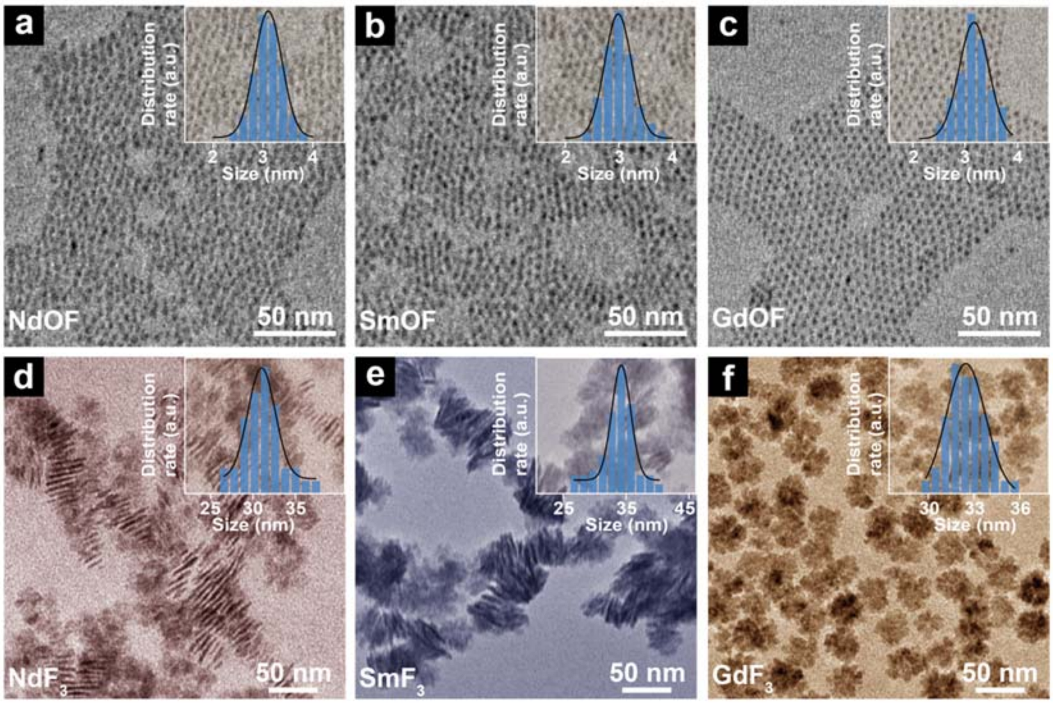

Supplementary Fig. 8 Characterization of different LnOF NPs and corresponding LnF_3 NCs. TEM images of **a** NdOF NPs, **b** SmOF NPs, **c** GdOF NPs, as well as **d** NdF_3 NCs, **e** SmF_3 NCs, and **f** GdF_3 NCs. Insets in **a-f** are statistics of size distribution of respective samples.

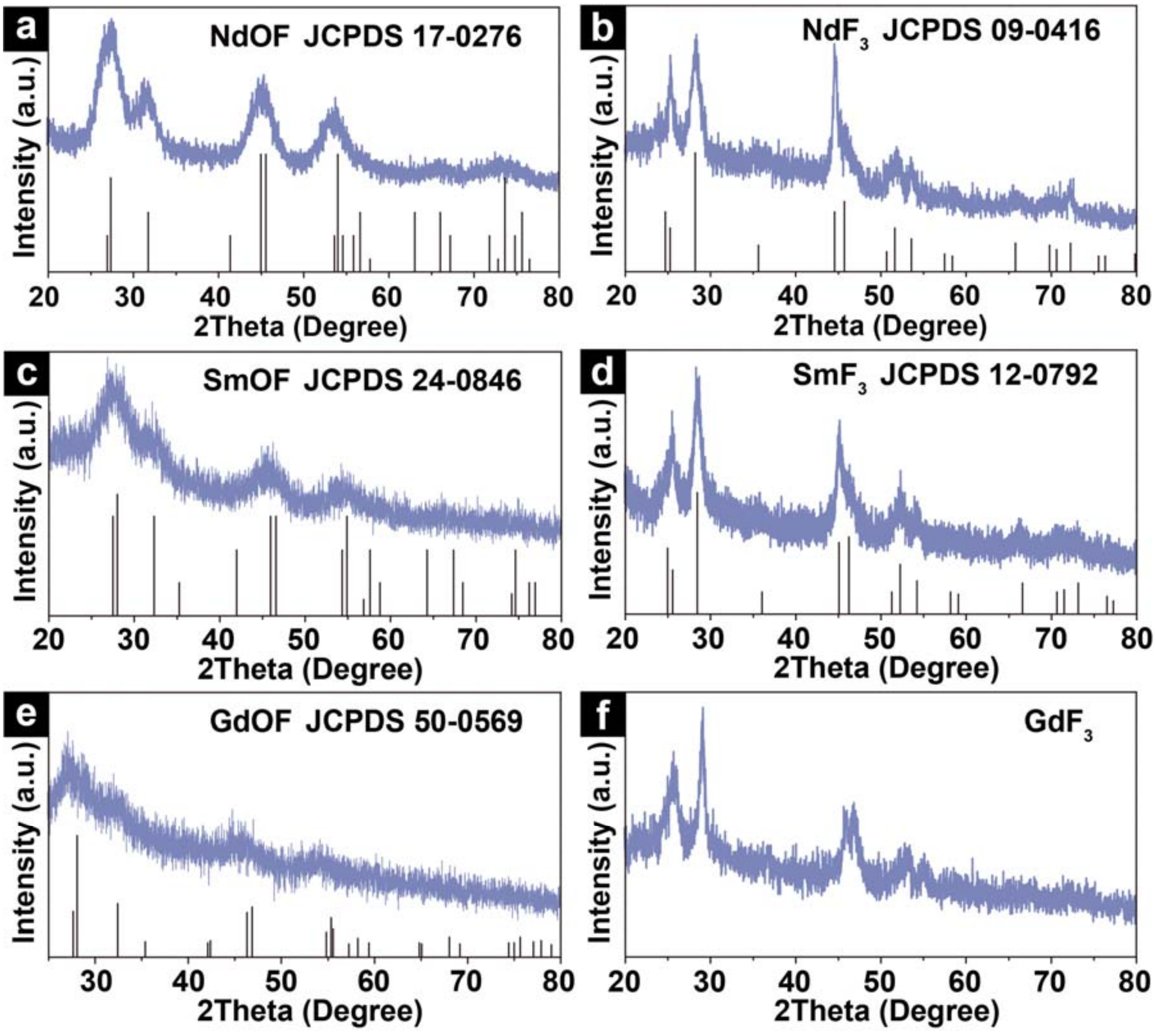

Supplementary Fig. 9 Crystal structure characterizations of different LnOF NPs and corresponding LnF_3 NCs. XRD patterns of NPs of **a** NdOF, **c** SmOF, and **e** GdOF. XRD patterns of NCs of **b** NdF_3, **d** SmF_3, and **f** GdF_3 [1].

Supplementary Table 1. Summary of experimental details for PL measurement.

Mode	λ/nm	Fixed Slit/nm	Scan Slit/nm
Excitation Spectra	$\lambda_{em} = 591$	1	1
Emission Spectra	$\lambda_{ex} = 273$	1.5	3
Emission Spectra	$\lambda_{ex} = 395$	0.8	2

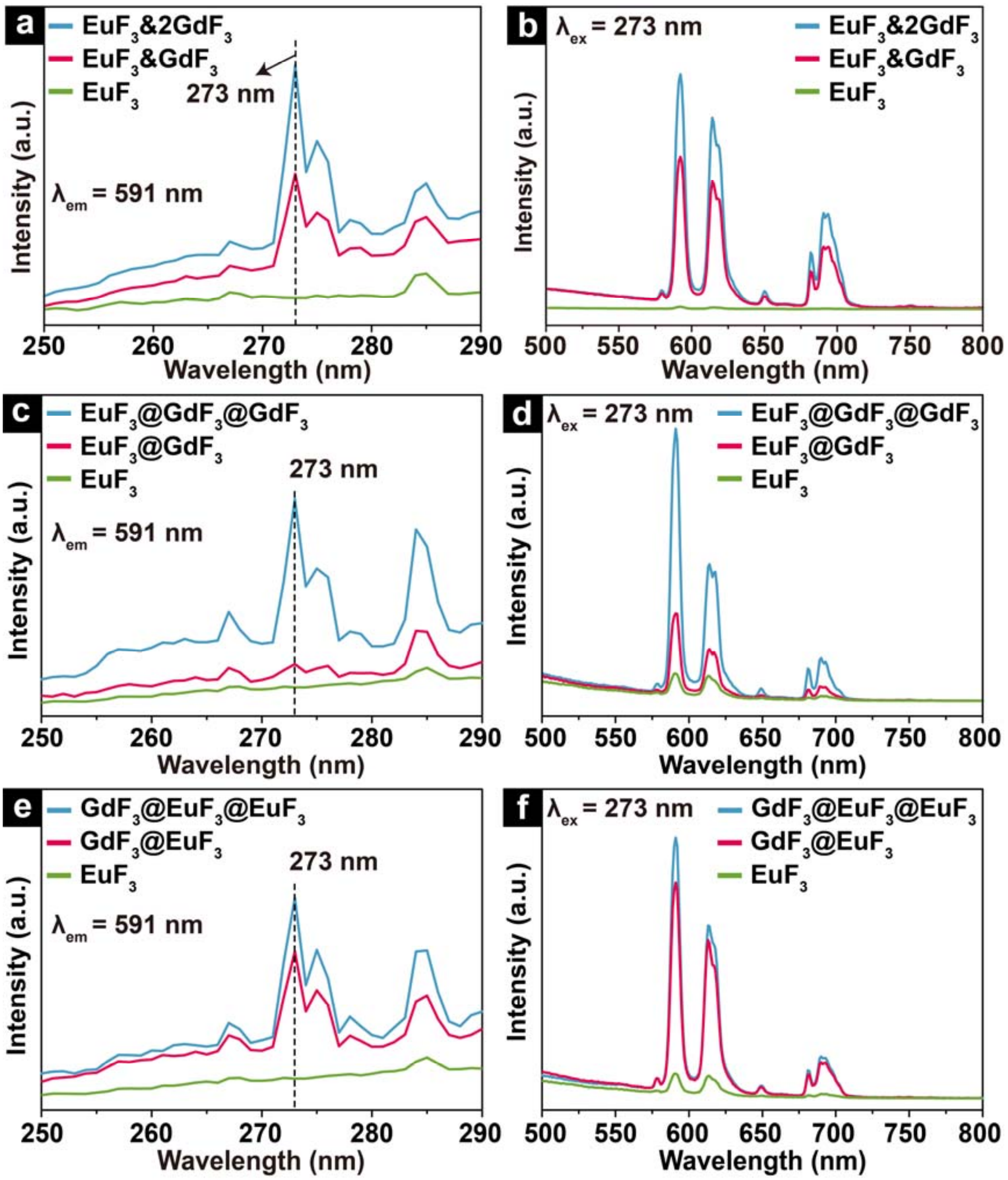

Supplementary Fig. 10 The PL evolution of Eu^{3+} in different NCs. **a** Excitation and **b** PL emission spectra of EuF_3&GdF_3 dimeric NCs with different GdF_3/EuF_3 molar ratios under the excitation of 273 nm. **c** Excitation and **d** PL emission spectra of EuF_3 NCs with different number of GdF_3 shells under the excitation of 273 nm. **e** Excitation and **f** PL emission spectra of GdF_3 NCs with different number of EuF_3 shells under the excitation of 273 nm.

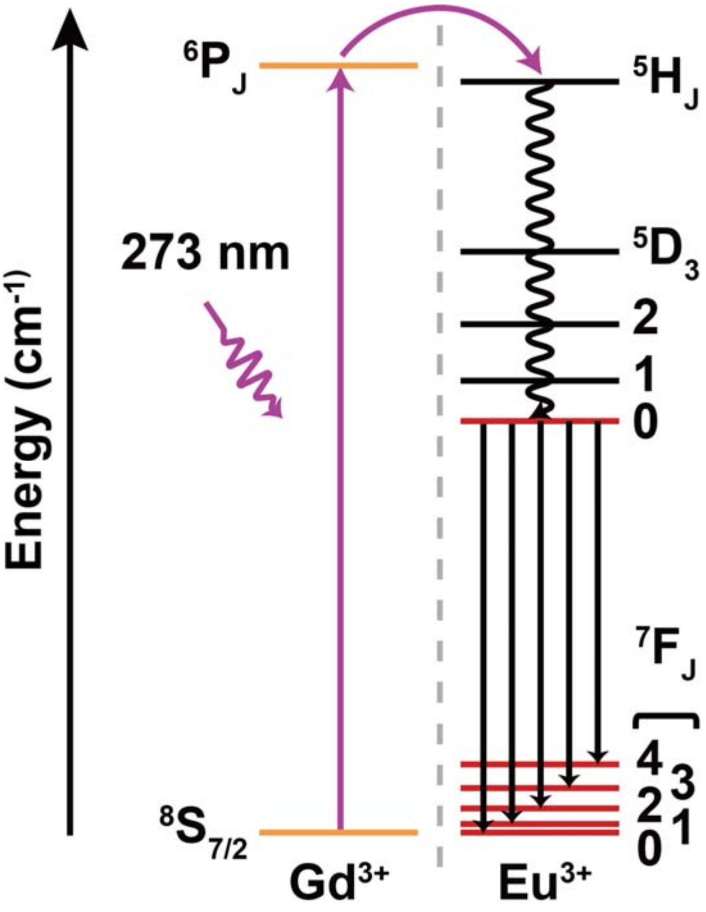

Supplementary Fig. 11 Gd^{3+}-Eu^{3+} energy transfer pathway. Energy level diagrams of the Gd^{3+}-Eu^{3+} energy transfer system, under direct excitation of Gd^{3+} using 273 nm light.

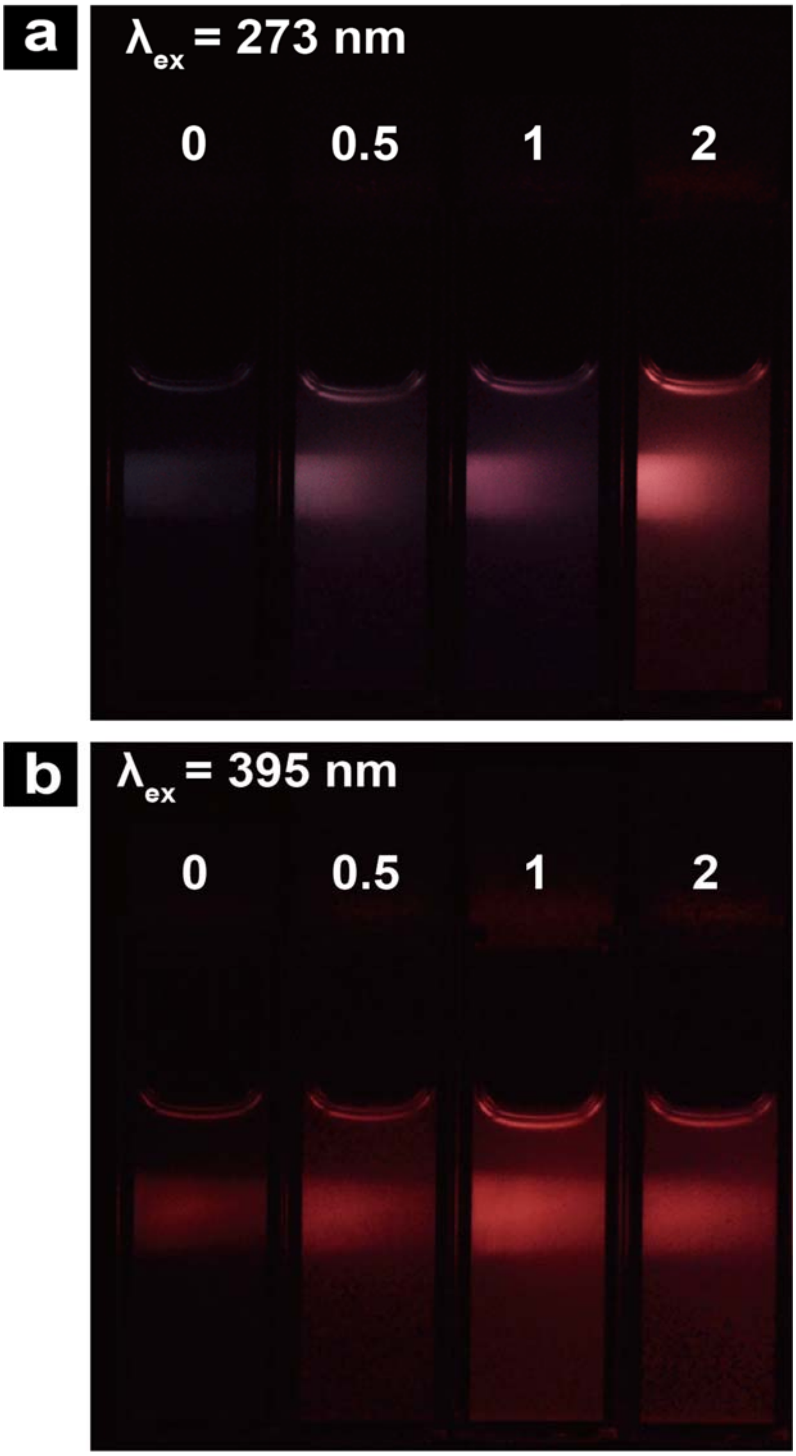

Supplementary Fig. 12 PL intensity variation of Eu^{3+} in different dimeric NCs. Digital optical images of ethanol solutions containing EuF_3&GdF_3 dimeric NCs in a cuvette with GdF_3/EuF_3 molar ratios varying from 0:1 to 2:1 under **a** 273 and **b** 395 nm light excitation, respectively.

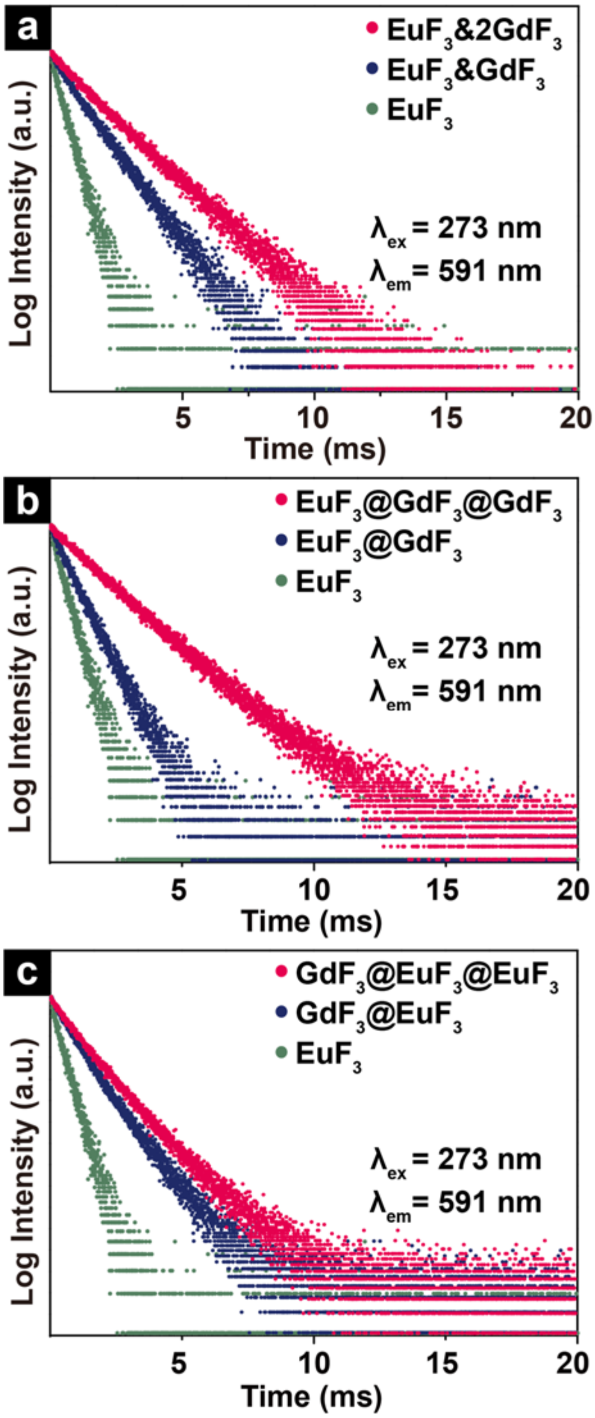

Supplementary Fig. 13 Luminescence decay curves of Eu^{3+} in different NCs. **a** Luminescence decay curves of EuF_3&GdF_3 dimeric NCs with different GdF_3/EuF_3 molar ratios. **b** Luminescence decay curves of Eu^{3+} in NCs with different number of GdF_3 shells. **c** Luminescence decay curves of Eu^{3+} in NCs with different number of EuF_3 shells. The working wavelength of Gd^{3+} at 273 nm was used as excitation source.

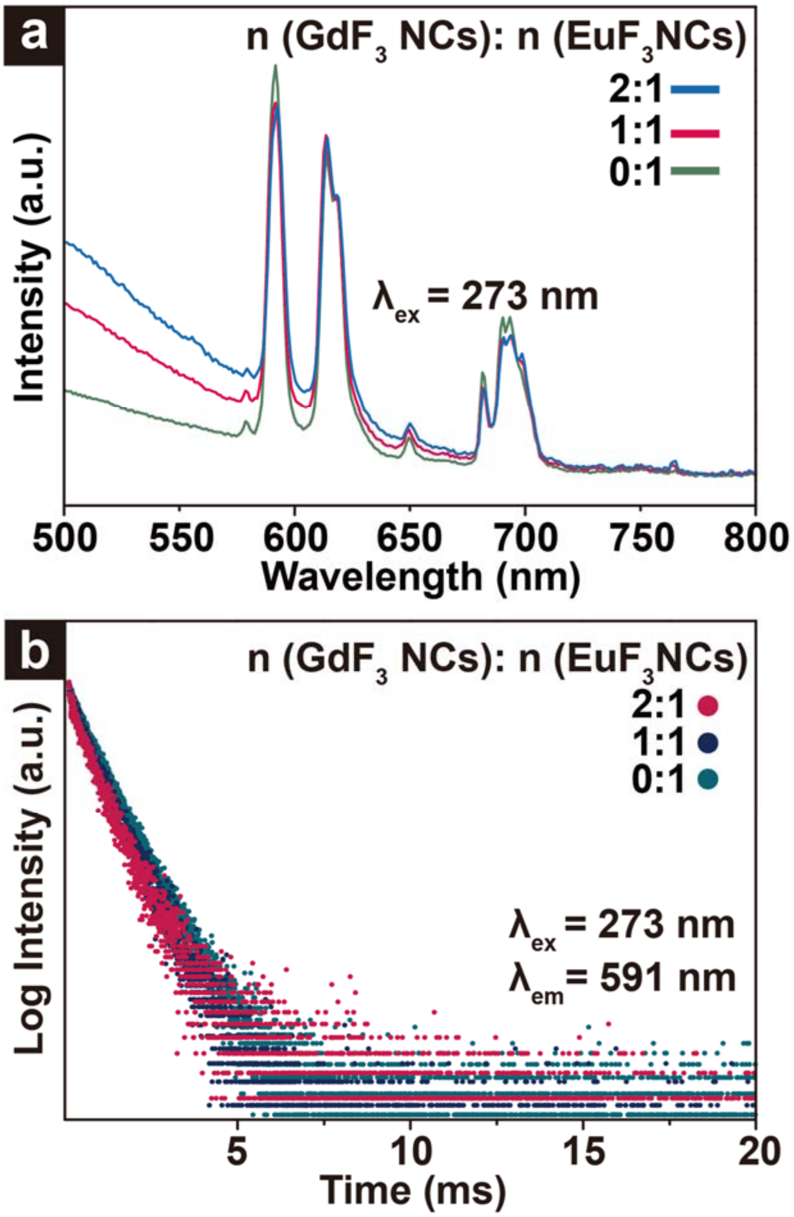

Supplementary Fig. 14 The PL properties of Eu^{3+} in mixed solutions of GdF_3 and EuF_3 NCs. **a** PL emission spectra and **b** decay curves of Eu^{3+} in physically mixed solutions of GdF_3 and EuF_3 NCs at different molar ratios under the excitation of 273 nm.

Supplementary Equation 1. Calculation of quantum yields.

To calculate the quantum yields (QYs) of EuF_3 component in dimeric, core-shelled and reversely core-shelled NCs, the intrinsic Eu^{3+} radiative lifetime (τ_c) of the $^5D_0$-^7F_J state was calculated by using the formula [2-3]:

$$1/\tau_c = A_{MD,0} \times n^3 \times \left(I_{tot}/I_{MD}\right)$$

where n is the refractive index of the solvent, equal to 1.36 (ethanol); $A_{MD,0}$ is the spontaneous emission probability for the $^5D_0$-$^7F_1$ transition in vacuum, equal to 14.65 s^{-1}; and (I_{tot}/I_{MD}) is the ratio of the total Eu^{3+} emission spectrum to the area of the $^5D_0 \rightarrow ^7F_1$ band. The τ_c of NCs with different structure are calculated and the corresponding QYs can be calculated from the observed luminescence lifetime (τ_{obs}):

$$\phi_{Eu} = \tau_{obs}/\tau_c$$

The calculated QYs are listed in Supplementary Table 2, which are consistent with emission intensity variation of Eu^{3+} in different NC structures. It's worth pointing out that under the excitation of 395 nm, the calculated QYs is the intrinsic emission quantum yield.

Supplementary Table 2. Calculated QYs of NCs in different structures.

Types of NCs	QYs/% (λ_{Ex} = 273 nm)	QYs/% (λ_{Ex} = 395 nm)
EuF_3	13.4	9.4
EuF_3&GdF_3	22.1	22.3
EuF_3&2GdF_3	29.3	31.6
EuF_3@GdF_3	15.8	10.8
EuF_3@GdF_3@GdF_3	25.6	21.6
GdF_3@EuF_3	16.0	15.1
GdF_3@EuF_3@EuF_3	17.9	16.2

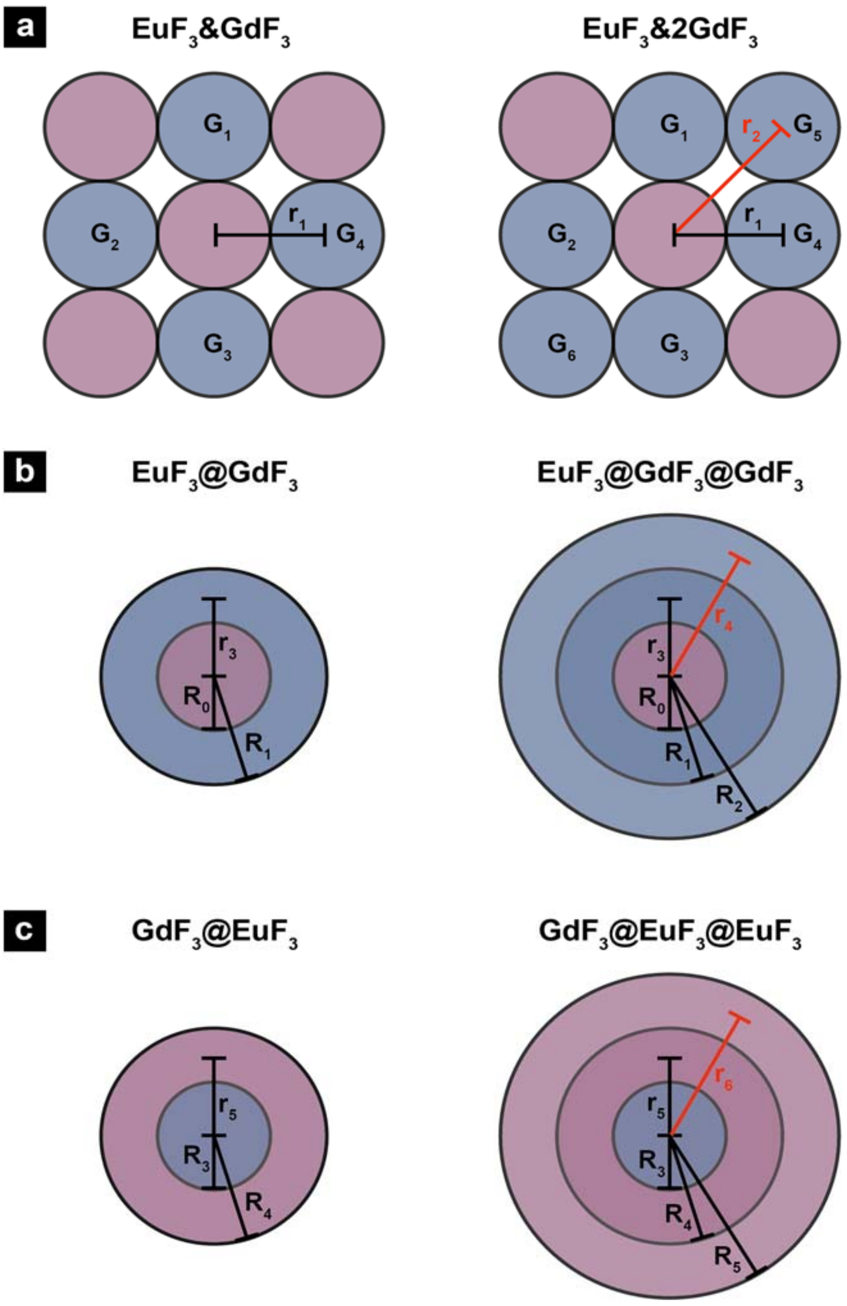

Supplementary Fig. 15 Calculation of energy transfer rate. The simplified models for energy transfer calculation in **a** EuF_3&GdF_3 and EuF_3&2GdF_3 dimeric, **b** EuF_3@GdF_3 and EuF_3@GdF_3@GdF_3 core-shelled, as well as **c** GdF_3@EuF_3 and GdF_3@EuF_3@EuF_3 reversely core-shelled NCs. EuF_3 and GdF_3 components are presented in light purple and grey blue respectively. r_i refers to the distance between donor (GdF_3 NPs) and acceptor (EuF_3 NPs), and R_i refers to the radius of different NCs.

We calculated the energy transfer rate in different photonic environments according to the following equation:

$$K_{ET} = \frac{c}{r^6}$$

where K_{ET} is the energy transfer rate from donor (GdF_3 NP) to acceptor (EuF_3 NP), c represents the "energy transfer strength" and is a constant in our system, r is the donor-acceptor distance [4, 5]. We would like to emphasize that the energy transfer rate here can also be understood as the energy acceptance rate of EuF_3 NPs from adjacent GdF_3 NPs.

However, those so far reported strategies are applied to calculate the energy transfer processes occurred between adjacent ions [5-7]. In our case, the donor (GdF_3) and acceptor (EuF_3) are separated NPs, and the connection among those individual NPs during self-assembly process makes it possible to realize efficient GdF_3-EuF_3 energy transfer. So, the modelling of our NC system is a bit complicated and the energy flux process including energy transfer, energy migration, and energy loss induced by concentration or solvent quenching must be considered at the same time. Even though we couldn't give a comprehensive calculation right now, we would like endeavor to give a precise-possible calculation based on rationally simplified models, in which the GdF_3-EuF_3 distances and the GdF_3/EuF_3 ratios are mainly concerned.

For dimeric NCs, the co-assembly of EuF_3 NPs and GdF_3 NPs undergoes completely. As a typical example, the calculated Eu/Gd atomic ratio of 33.3:66.7 from elemental mapping result confirmed the EuF_3&$2GdF_3$ composition of the dimeric structure, which perfectly matches with our experimentally designs of respective NCs and suggests the perfect and complete self-assembly of EuF_3 and GdF_3 NPs. So, every single EuF_3 NPs in

EuF_3&2GdF_3 structure will accept more energy from surrounding GdF_3 NPs than in the case of EuF_3&GdF_3 due to the increased GdF_3/EuF_3 ratio.

As shown in Supplementary Fig. 15a, the energy transfer rate from adjacent GdF_3 NPs to a single EuF_3 NP in EuF_3&GdF_3 (K_{sum1}) and EuF_3&2GdF_3 (K_{sum2}) can be calculated as follows:

$$K_{sum1} = \sum_{i=1}^{4} K_i = K_1 + K_2 + K_3 + K_4 = \frac{4c}{r_1^6}$$

$$K_{sum2} = \sum_{i=1}^{6} K_i = K_1 + K_2 + \cdots + K_6 = \frac{4c}{r_1^6} + \frac{2c}{r_2^6}$$

$$\frac{K_{sum2}}{K_{sum1}} = 1 + \frac{r_1^6}{2r_2^6}$$

where r_1 = 3 nm referring to the sum of the radii of EuF_3 and GdF_3 NPs, and the subsequently calculated value of r_2 is 4.24 nm (Supplementary Fig. 15a), c as a constant can be reduced. The calculated K_{sum2}/K_{sum1} = 106%, suggesting the increased energy transfer rate in EuF_3&2GdF_3 compared with that in EuF_3&GdF_3. However, this value is smaller than the calculated increase of QYs: QYs_2/QYs_1 = 132%, which is reasonable because EuF_3&GdF_3 may suffer from more severe concentration quenching caused by cross relaxation between EuF_3 NPs, due to the relatively low content of GdF_3 compared with that in EuF_3&2GdF_3. So, the increased QYs benefited from not only the enhanced energy transfer efficiency but also the inhibition of concentration quenching among EuF_3 NPs due to the spatial separation of GdF_3 NPs. Thus, it is necessary to introduce a correction factor (α) to refine the final energy transfer rate and QYs:

$$\alpha \frac{K_{sum2}}{K_{sum1}} = \frac{QYs_2}{QYs_1} (\alpha > 1)$$

For core-shelled $EuF_3@GdF_3$ and $EuF_3@GdF_3@GdF_3$ structures, the inner EuF_3 NCs core and outer GdF_3 NCs shell can be treated as an integral whole acceptor and donor respectively, and r is determined by the distance from geometric center of EuF_3 core and GdF_3 shell. As shown in Supplementary Fig. 15b, the energy transfer rate for EuF_3 NCs core in $EuF_3@GdF_3$ (K_{sum3}) and $EuF_3@GdF_3@GdF_3$ (K_{sum4}) can be calculated as follows:

$$K_{sum3} = \frac{c}{r_3^6}$$

$$K_{sum4} = \sum_{i=1}^{2} K_i = \frac{c}{r_3^6} + \frac{c}{r_4^6} \cdot \frac{\pi R_2^2 - \pi R_1^2}{\pi R_1^2 - \pi R_0^2}$$

$$\frac{K_{sum4}}{K_{sum3}} = 1 + \left(\frac{r_3}{r_4}\right)^6 \cdot \frac{\pi R_2^2 - \pi R_1^2}{\pi R_1^2 - \pi R_0^2}$$

in which r_3 = 23.9 nm refers to the sum of the radius of EuF_3 NCs and half of the thickness of the first GdF_3 shell, r_4 refers to the sum of the radius of $EuF_3@GdF_3$ NCs and half thickness of the second GdF_3 shell, which is 33.0 nm, and similarly the calculated value of R_0, R_1, and R_2 is 20.1, 27.7, and 38.3 nm respectively, c as a constant can be reduced. The calculated K_{sum4}/K_{sum3} = 128%, which shows obviously increased energy transfer rate in $EuF_3@GdF_3@GdF_3$ than in $EuF_3@GdF_3$. However, this value is smaller than the calculated increase of QYs: QYs_4/QYs_3 = 162%, for that the increased QYs benefited from not only the enhanced energy transfer efficiency but also the inhibition of solvent quenching provided by GdF_3 shell. It is also necessary to introduce a correction factor (β) here to refine the final energy transfer rate:

$$\beta \frac{K_{sum4}}{K_{sum3}} = \frac{QYs_4}{QYs_3} \ (\beta > 1)$$

Similarly, the energy transfer rate from GdF_3 core to EuF_3 shell in $GdF_3@EuF_3$ and $GdF_3@EuF_3@EuF_3$ reversely core-shell structured NCs can be calculated as following (Supplementary Fig. 15c):

$$K_{sum5} = \frac{c}{r_5^6}$$

$$K_{sum6} = \sum_{i=1}^{2} K_i = \frac{c}{r_5^6} + \frac{c}{r_6^6} \cdot \frac{\pi R_5^2 - \pi R_4^2}{\pi R_4^2 - \pi R_3^2}$$

$$\frac{K_{sum6}}{K_{sum5}} = 1 + \left(\frac{r_5}{r_6}\right)^6 \cdot \frac{\pi R_5^2 - \pi R_4^2}{\pi R_4^2 - \pi R_3^2}$$

where similarly as above, the calculated value of r_5, r_6 is 19.9 and 27.0 nm, respectively, while those of R_3, R_4, and R_5 are 16.9, 22.9, and 31.1 nm, respectively, *c* as a constant can be reduced. The calculated K_{sum6}/K_{sum5} = 130%, which shows obviously increased energy transfer rate in $GdF_3@EuF_3@EuF_3$ than in $GdF_3@EuF_3$. However, this value is larger than the increased QYs: QYs_6/QYs_5 = 112%. This is also reasonable because although more EuF_3 NPs in $GdF_3@EuF_3@EuF_3$ receive energy from GdF_3 core, which enhances the energy transfer efficiency, the energy transfer efficiency is also suffered from enhanced concentration quenching among EuF_3 component. Moreover, since the energy transferred from Gd^{3+} is fixed, the second layer of Eu^{3+} can only absorb the residual energy that passes through the first Eu^{3+} layer, and the relatively long distance from GdF_3 core to EuF_3 shell also hinders the energy transfer. So, a correction factor (γ) is introduced here to refine the final energy transfer rate:

$$\gamma \frac{K_{sum6}}{K_{sum5}} = \frac{QYs_6}{QYs_5} \ (0<\gamma<1)$$

According to the above calculations, we can see that the increase of energy transfer rate occurs in all the dimeric, core-shelled, and reversely core-shelled NCs, which is consistent with our experimental data. However, it's worth pointing out that due to the contributions from the variation of energy loss processes including concentration and solvent quenching, the correction factor, α, β, and γ mentioned above, shall be considered to refine the model system.

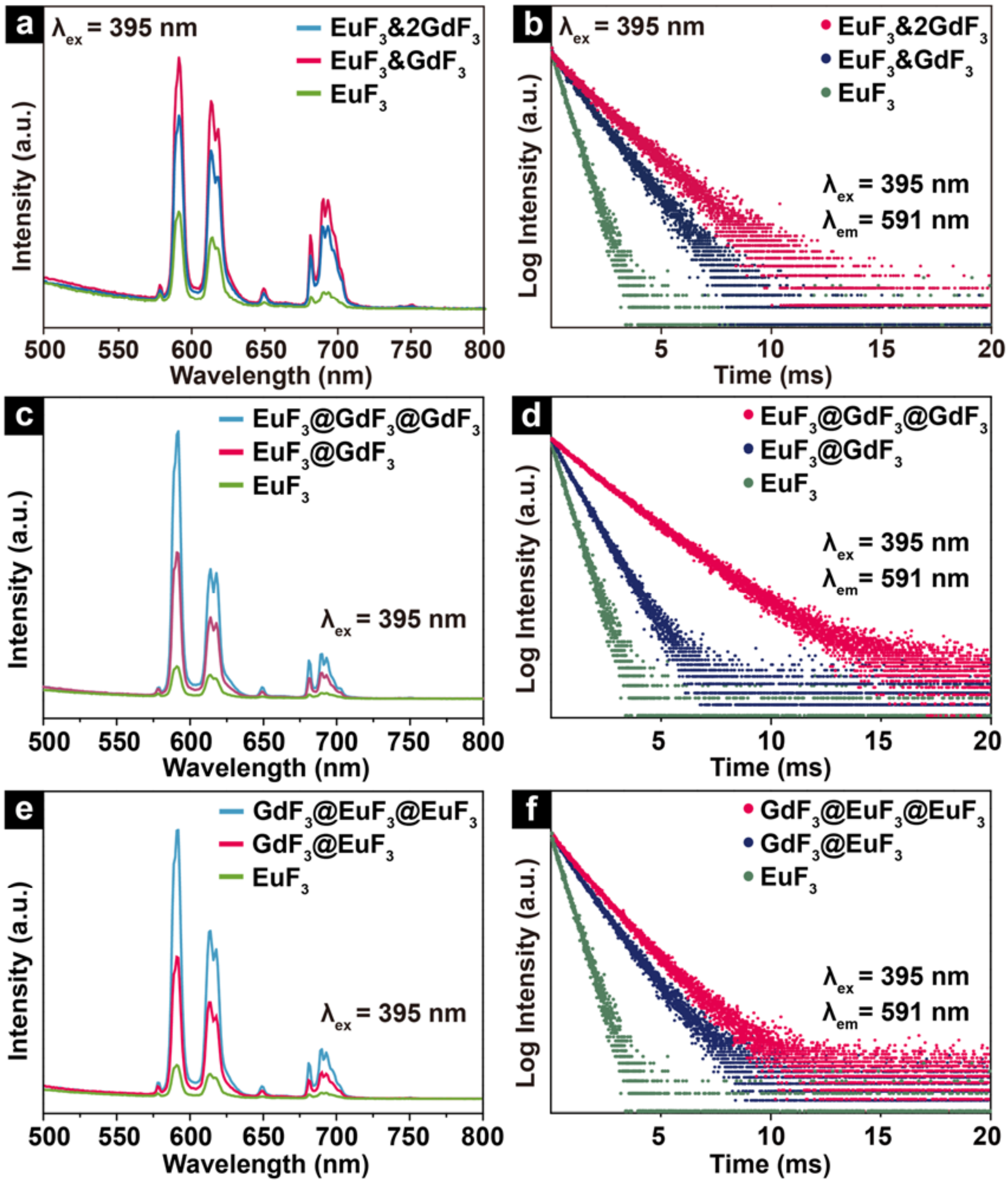

Supplementary Fig. 16 The PL evolution of Eu^{3+} in different NCs. **a** PL emission spectra and **b** luminescence decay curves of Eu^{3+} in EuF_3&GdF_3 dimeric NCs with different GdF_3/EuF_3 molar ratios. **c** PL emission spectra and **d** luminescence decay curves of Eu^{3+} in NCs of EuF_3, EuF_3@GdF_3, and EuF_3@GdF_3@GdF_3. **e** PL emission spectra and **f** luminescence decay curves of Eu^{3+} in NCs of EuF_3, GdF_3@EuF_3, and GdF_3@EuF_3@EuF_3. The working wavelength of Eu^{3+} at 395 nm was used as excitation source.

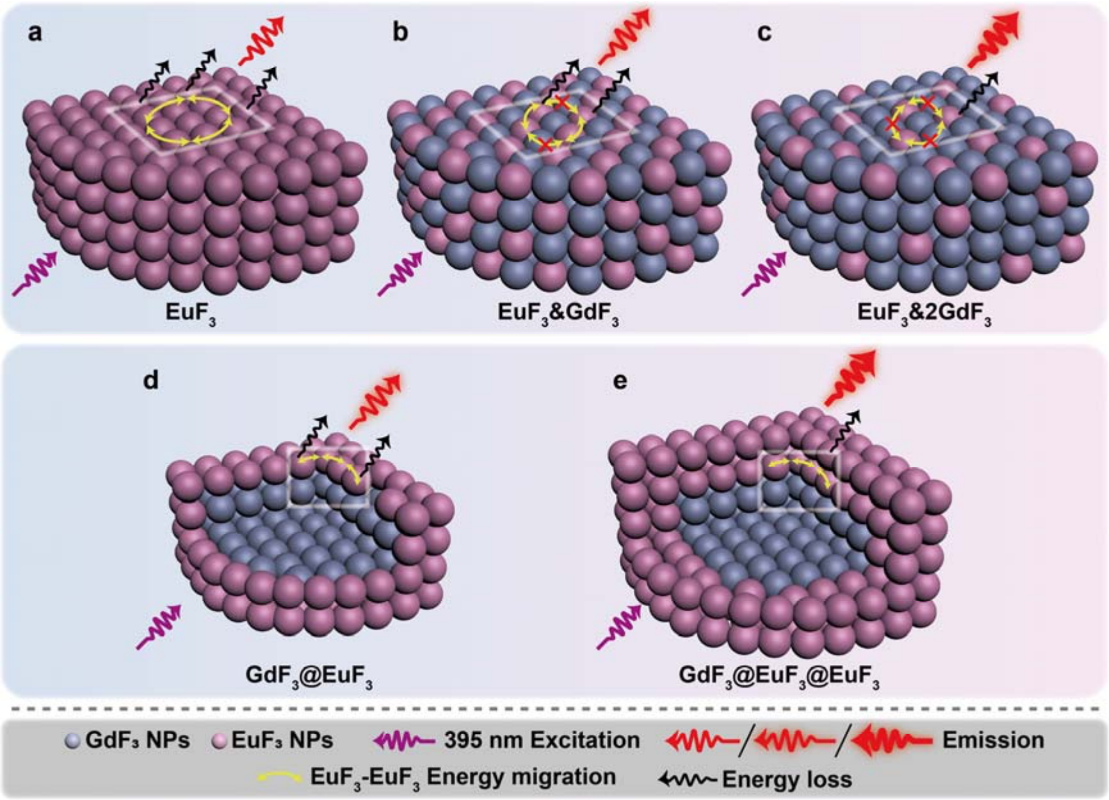

Supplementary Fig. 17 Schematic illustration of the energy migration of Eu^{3+} in different NCs. The energy migration among Eu^{3+}-Eu^{3+} ion pairs as well as subsequent luminescence emission and energy loss in NCs of **a** EuF_3, **b** EuF_3&GdF_3, **c** EuF_3&2GdF_3, and core-shelled NCs of **d** GdF_3@EuF_3 and **e** GdF_3@EuF_3@EuF_3. The working wavelength of Eu^{3+} at 395 nm was used as excitation source.

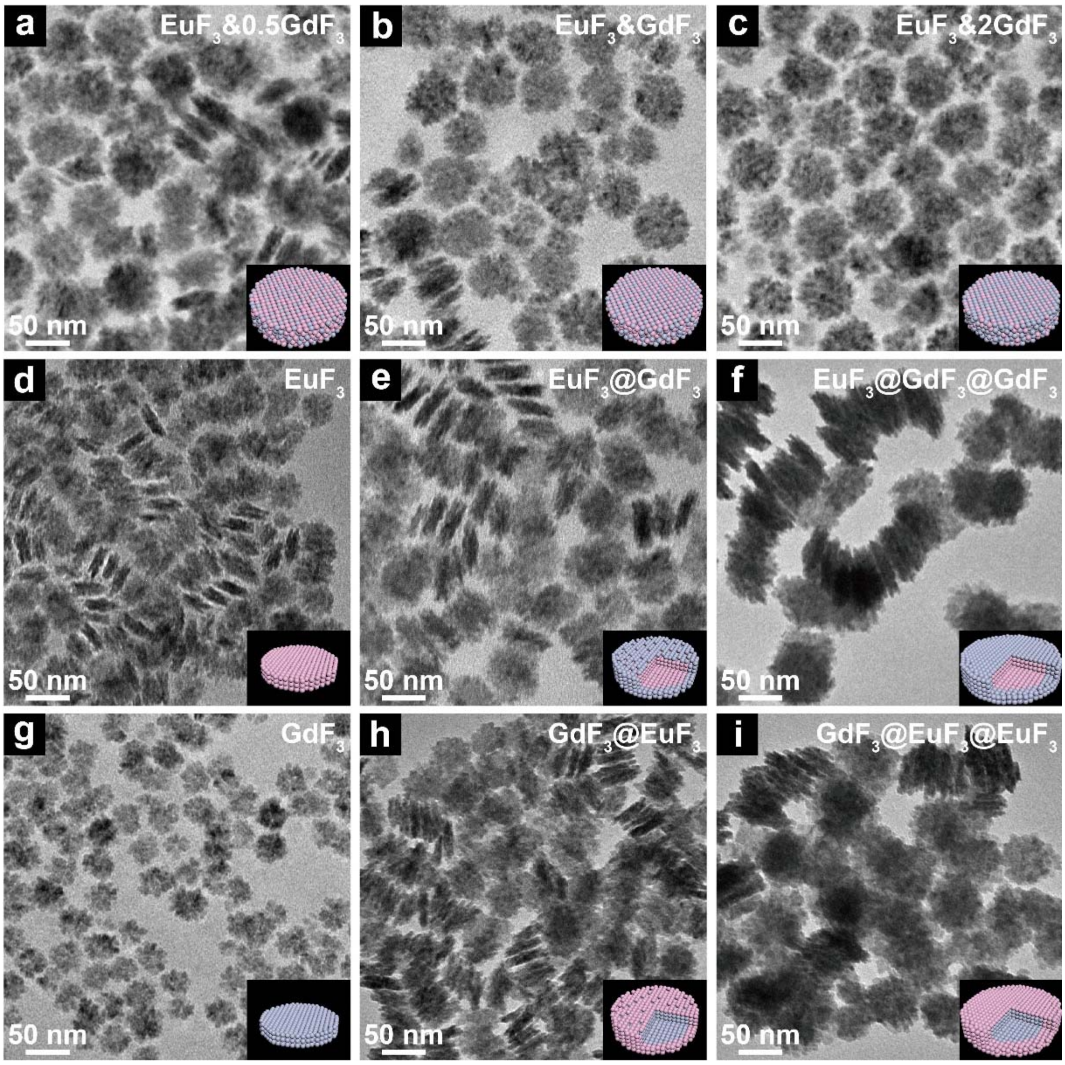

Supplementary Fig. 18 Morphology characterization of different-structured NCs. TEM images of dimeric NCs of **a** EuF_3&0.5GdF_3, **b** EuF_3&GdF_3, and **c** EuF_3&2GdF_3. TEM images of core-shelled NCs of **d** EuF_3, **e** EuF_3@GdF_3, and **f** EuF_3@GdF_3@GdF_3. TEM images of reversely core-shelled NCs of **g** GdF_3, **h** GdF_3@EuF_3, and **i** GdF_3@EuF_3@EuF_3.

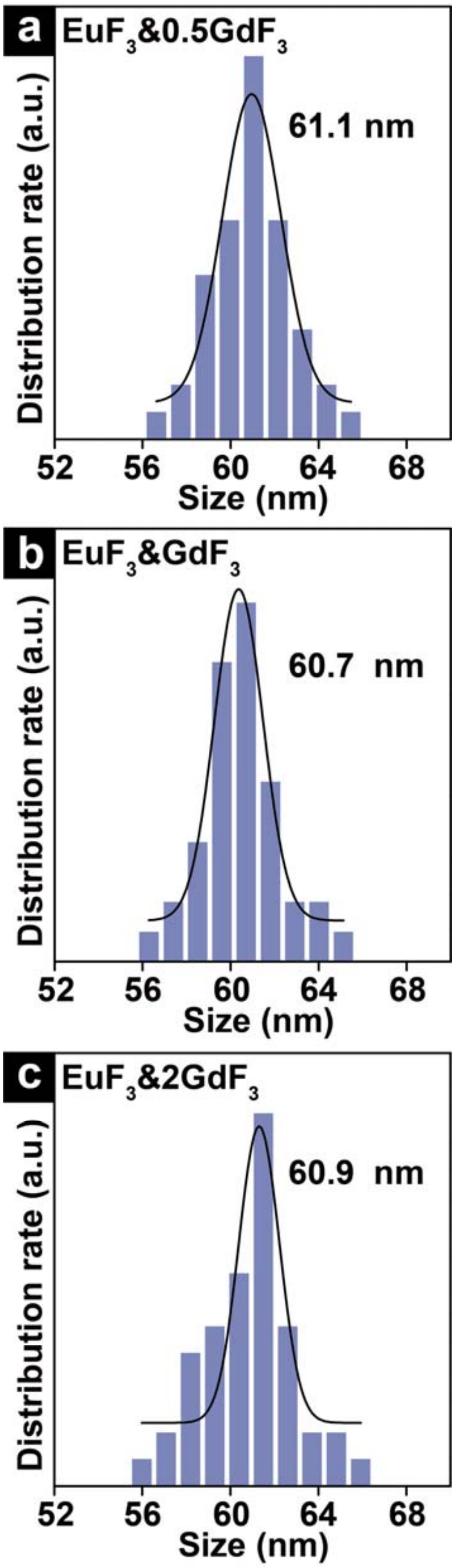

Supplementary Fig. 19 Calculated size distribution of different dimeric NCs. Statistics of size distributions of dimeric NCs of **a** EuF_3&0.5GdF_3, **b** EuF_3&GdF_3, and **c** EuF_3&2GdF_3.

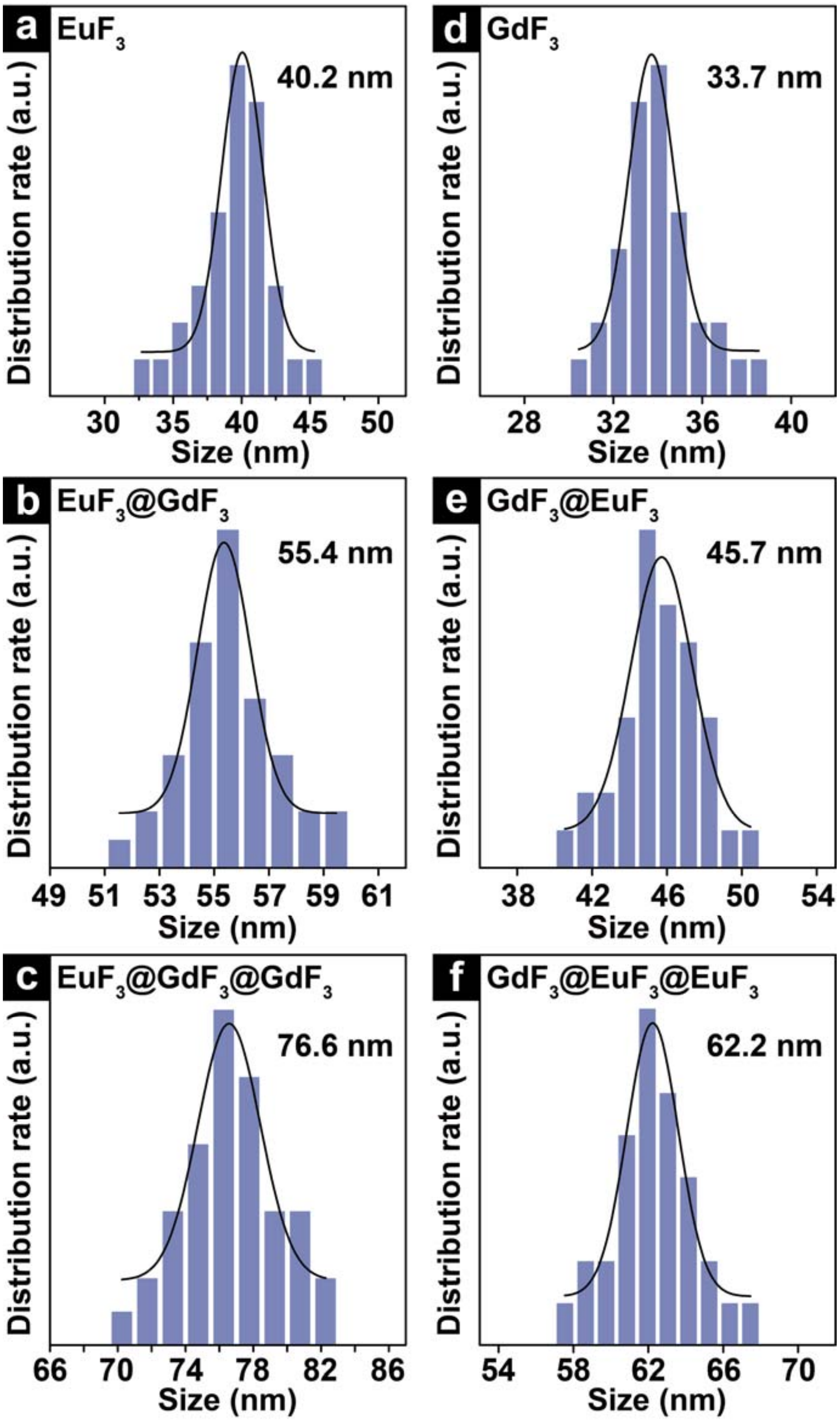

Supplementary Fig. 20 Calculated size distribution of different core-shelled NCs. Statistics of size distributions of core-shelled NCs of **a** EuF_3, **b** $EuF_3@GdF_3$, and **c** $EuF_3@GdF_3@GdF_3$. Statistics of size distributions of reversely core-shelled NCs of **d** GdF_3, **e** $GdF_3@EuF_3$, and **f** $GdF_3@EuF_3@EuF_3$.

Supplementary References

1. Xiao, S. *et al*. From Trifluoroacetate complex precursors to monodisperse Rare-Earth fluoride and Oxyfluoride nanocrystals with diverse shapes through controlled fluorination in solution phase. *Chem. Eur. J.* **13**, 2320-2332 (2007).

2. Du, Y. et al. Highly luminescent self-organized sub-2-nm EuOF nanowires. *J. Am. Chem. Soc*. **131**, 16364-16365 (2009).

3. Werts, M. H. V., Jukes, R. T. F. & Verhoeven, J. W. The emission spectrum and the radiative lifetime of Eu^{3+} in luminescent lanthanide complexes. *Phys. Chem. Chem. Phys*. **4**, 1542-1548 (2002).

4. Forster, T. Zwischenmolekulare Energiewanderung und Fluoreszenz. *Ann. Phys*. **437**, 55-75 (1948).

5. Rabouw, F. T., Den Hartog, S. A., Senden, T. & Meijerink, A. Photonic effects on the Forster resonance energy transfer efficiency. *Nat. Commun*. **5**, 3610 (2014).

6. Omagari, S. et al. Critical role of energy transfer between terbium ions for suppression of back energy transfer in nonanuclear terbium clusters. *Sci. Rep*. **6**, 37008 (2016).

7. Kasprzycka, E. Contribution of energy transfer from the singlet state to the sensitization of Eu^{3+} and Tb^{3+} luminescence by Sulfonylamidophosphates. *Chem. Eur. J.* **23**, 1318-1330 (2017).
